# HIV-1 latency and virus production from unintegrated genomes following direct infection of resting CD4 T cells

**DOI:** 10.1186/s12977-015-0234-9

**Published:** 2016-01-05

**Authors:** Chi N. Chan, Benjamin Trinité, Caroline S. Lee, Saurabh Mahajan, Akanksha Anand, Dominik Wodarz, Steffanie Sabbaj, Anju Bansal, Paul A. Goepfert, David N. Levy

**Affiliations:** Department of Basic Science, New York University College of Dentistry, New York, NY 10010 USA; Department of Ecology and Evolutionary Biology, University of California, Irvine, School of Biological, Sciences, Irvine, CA 92697 USA; Department of Medicine, University of Alabama at Birmingham, Birmingham, AL 35294 USA

**Keywords:** HIV-1, Latency, Unintegrated DNA, Raltegravir, Integration, Cytotoxic T cells

## Abstract

**Background:**

HIV-1 integration is prone to a high rate of failure, resulting in the accumulation of unintegrated viral genomes (uDNA) in vivo and in vitro. uDNA can be transcriptionally active, and circularized uDNA genomes are biochemically stable in non-proliferating cells. Resting, non-proliferating CD4 T cells are prime targets of HIV-1 infection and latently infected resting CD4 T cells are the major barrier to HIV cure. Our prior studies demonstrated that uDNA generates infectious virions when T cell activation follows rather than precedes infection.

**Results:**

Here, we characterize in primary resting CD4 T cells the dynamics of integrated and unintegrated virus expression, genome persistence and sensitivity to latency reversing agents. Unintegrated HIV-1 was abundant in directly infected resting CD4 T cells. Maximal gene expression from uDNA was delayed compared with integrated HIV-1 and was less toxic, resulting in uDNA enrichment over time relative to integrated proviruses. Inhibiting integration with raltegravir shunted the generation of durable latency from integrated to unintegrated genomes. Latent uDNA was activated to de novo virus production by latency reversing agents that also activated latent integrated proviruses, including PKC activators, histone deacetylase inhibitors and P-TEFb agonists. However, uDNA responses displayed a wider dynamic range, indicating differential regulation of expression relative to integrated proviruses. Similar to what has recently been demonstrated for latent integrated proviruses, one or two applications of latency reversing agents failed to activate all latent unintegrated genomes. Unlike integrated proviruses, uDNA gene expression did not down modulate expression of HLA Class I on resting CD4 T cells. uDNA did, however, efficiently prime infected cells for killing by HIV-1-specific cytotoxic T cells.

**Conclusions:**

These studies demonstrate that contributions by unintegrated genomes to HIV-1 gene expression, virus production, latency and immune responses are inherent properties of the direct infection of resting CD4 T cells. Experimental models of HIV-1 latency employing directly infected resting CD4 T cells should calibrate the contribution of unintegrated HIV-1.

**Electronic supplementary material:**

The online version of this article (doi:10.1186/s12977-015-0234-9) contains supplementary material, which is available to authorized users.

## Background

The failure of the majority of HIV-1 reverse transcripts to integrate into cellular chromosomal DNA has been apparent since the initial molecular descriptions of infection in vivo and in vitro [1, 2] (reviewed in [3, 4]). Unintegrated HIV-1 DNA (uDNA) can be transcriptionally active, and production of early viral RNA and proteins has been observed in several cell types including resting CD4 T cells [5, 6]. The number of transcriptionally active uDNA genomes can rival the number of active integrated proviruses [7]. Integration-defective lentiviral vector systems exploit this transcriptional activity, as well as biochemical stability of circularized extrachromosomal genomes in non-replicating cells [8–11] to achieve sustained ectopic gene expression in multiple lineages in vitro and in vivo (reviewed in [12]). uDNA gene expression is higher in non-proliferating cells compared with proliferating cells such as transformed cell lines and activated CD4 T cells, perhaps owing to a lack of dilution of uDNA templates and their RNA and protein products [4, 13].

We have previously reported that when cellular coinfection places an integrated provirus (iDNA) in a cell together with an unintegrated genome, viral complementation allows completion of the unintegrated virus’ replication cycle without integration [14]. Recently, we reported that when HIV-1 infects resting T cells several days prior to T cell activation, uDNA alone generates infectious virions [6]. This stands in contrast to infection of activated T cells or cell lines, which support only transient and reduced levels of gene expression from uDNA without de novo virus production. Also in contrast to activated T cells and cell lines, gene expression from uDNA in resting T cells required delivery of virion-associated Vpr, evidence of the uniqueness of uDNA gene regulation. The sequence of events which we found to induce production of infectious virions from uDNA mimics an in vivo situation where an infected resting CD4 T cell is activated following migration to lymphoid tissues, which has been proposed as a mechanism facilitating the establishment of HIV-1 infection [15–17].

The predominant model for establishment of HIV-1 latency entails the infection of an activate CD4 T cell that returns to a resting state, thus removing support for viral transcription [18]. On the other hand, during acute and early infection, resting CD4 T cells are frequent targets of infection, constituting up to 90 % of viral RNA+ cells in both HIV-1 infected humans and SIV infected macaques [19, 20]. The latent reservoir is also rapidly established during acute infection [21–23], supporting the notion that direct infection of resting CD4 T cells may also contribute to the latent reservoir [19, 24, 25]. The majority of infected cells during untreated chronic infection also contain only unintegrated HIV–1 DNA [26]. Although unstimulated peripheral blood T cells are resistant to infection in vitro [27], in vivo, HIV-1 replicates in lymphoid (LT) and mucosal tissues that provide microenvironmental factors (cytokines, chemokines, DC and stromal cells) that support HIV-1 replication and maintain cell viability [28, 29]. Several important in vitro models of HIV-1 latency utilize these factors to promote survival of resting CD4 T cells in vitro and to increase their permissiveness to infection by HIV-1 [30]. These systems are extensively utilized to test the response of latent HIV-1 to various compounds under scrutiny as latency reversing agents for curative therapies.

To this end, we recently described a convenient and relevant model system [6] in which resting peripheral blood CD4 T cells are treated with common gamma chain cytokines such as IL-4 that render them permissive to infection without inducing cell activation, though it is known that common gamma chain cytokines induce signaling pathways such as stat5 or stat6 and increase the expression of the survival protein Bcl-2 [31, 32]. IL-4, which we employ here, has been implicated in facilitating HIV-1 replication in lymphoid tissues [33] and, as a product of various immune cells including T cells [34], assists HIV transcription [35]. Recently we have shown that the primary function of primary gamma chain cytokines in assisting infection of resting CD4 T cells in vitro is to prevent HIV-1-induced cell death early after infection that is triggered by reverse transcription and virion Vpr, rather than to enhance specific replicative processes [36]. With this system, we observed that latency persisted in resting CD4 T cells for several weeks, after which de novo virus production could be elicited by T cell activation. Importantly, using either the integrase inhibitor raltegravir or class I integrase mutants, we observed that durable, reversible latency was efficiently established by unintegrated HIV-1. Neither stimulation of cell activation nor virus expression induced proviral integration in this system. Virus production from uDNA was about one order of magnitude lower than from integrated proviruses but per-virion, was equally infectious [6].

In the present study we investigated the properties of uDNA and integrated proviruses (iDNA) in resting CD4 T cells, finding substantial and unexpected differences in the kinetics of their gene expression, persistence and virus production. We compared the responsiveness of latent unintegrated and latent integrated HIV-1 to a series latency reversing agents. Finally, we investigated the capacity of uDNA to down modulate Class I HLA expression and to prime infected cells for CTL killing. We conclude that unintegrated HIV-1 should be accounted for in models employing direct infection of non-proliferating cells such as resting CD4 T cells. Should these mechanisms pertain in vivo, the persistence of non-proliferating cells expressing HIV-1 proteins from uDNA may be threatened more by anti-HIV immunity than by biochemical degradation or viral cytopathic effects.

## Results

### Kinetics of gene expression by integrating and non-integrating HIV-1 in resting CD4 T cells

This study utilizes envelope-defective single round GFP reporter viruses expressing GFP in place of Nef, with Nef expressed at wild type levels downstream of an IRES element [37]. GFP fluorescence is generated during both the early and late gene expression phases, providing a sensitive measurement of overall HIV-1 gene expression that is highly correlated with virus production [6, 38]. To examine the properties of unintegrated genomes, integration was inhibited either with raltegravir (RAL) or by mutation of HIV-1 integrase at the catalytic domain [39], which we have previously demonstrated are functionally equivalent [6, 14], inhibiting integration by at least 2–3 orders of magnitude.

**Fig. 1:**
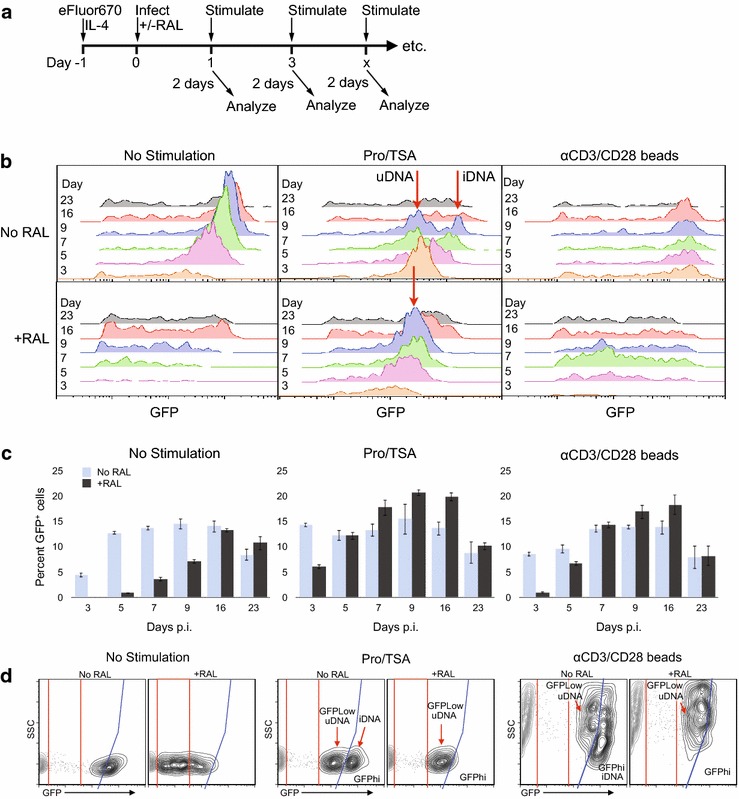
Kinetics of responsiveness to activating agents following initial infection of resting CD4 T cells. **a** Experimental design. After infection, samples of the IL-4-treated resting CD4 T cells were maintained in culture with or without raltegravir (RAL) for the indicated time period and were then stimulated by αCD3/CD28 activation beads or prostratin plus trichostatin (Pro/TSA) for 2 days before analysis by flow cytometry. IL-4 was replenished every 7 days. Raltegravir was added on the day of infection and on days 3, 7, 14 and 21. **b** Emergence of GFP+ productively infected cells. Pro/TSA or αCD3/CD28 beads were added 2 days prior to analysis. Only the GFP+ cells are displayed, and the areas under the curves represent the number of GFP+ cells present in each sample. Red arrows indicate subpopulations which we have previously shown to contain predominantly unintegrated HIV-1 (uDNA) or to contain at least one copy of integrated HIV-1 DNA (iDNA) [6]. One representative of 5 experiments is shown. Additional file 1: Fig. S1A in presents a similar experiment performed using an Int-D116N integrase active site mutant. **c** Percentage of cells that were GFP+. Experiment was performed in triplicate. To account for proliferation of GFP-negative cells, %GFP+ was calculated as the number of GFP+ cells divided by the number of total live cells, which was adjusted for proliferation using the Expansion Index in the Proliferation Platform of FlowJo 9. Cell divisions were measured by eFluor670 dilution. Typically ≤16 % of cells underwent division through day 14, with essentially all of them GFP-negative (Additional file 1: Fig. S4A). **d** Cell populations predominantly expressing HIV-1 from unintegrated (GFPlow) or integrated HIV-1 (GFPhi) can be parsed in αCD3/CD28 activated T cells by accounting for cellular heterogeneity (side scatter [SSC] profile). Equivalent results were obtained utilizing the Int-D116N active site mutant (Additional file 1: Fig S1B)

Given the long life span of resting CD4 T cells and the stability of circularized HIV-1 uDNA in them, we investigated the kinetics of gene expression from uDNA vs. iDNA in these cells after a single round of infection (Fig. 1). In the absence of further stimulation, integration-competent HIV-1 (No RAL) attained peak numbers of productively infected (GFP+) cells 7–9 days post infection (p.i.), consistent with studies demonstrating slowed kinetics of infection processes in resting vs. activated T cells [27, 40]. Unintegrated HIV-1 (+RAL) displayed even slower expression kinetics, with expression remaining very low during the first 7–9 days, consistent with prior studies examining short term infections [41]. uDNA expression peaked around 2 weeks after infection, and parallel results were obtained using a class I integrase mutant (Additional file 1: Fig. S1A). At peak expression, nearly as many GFP+ cells were generated from unintegrated as from integration-competent HIV-1 (Fig. 1c), though uDNA GFP MFI was four fold lower (Additional file 1: Fig. S2A) and HIV-1 RNA levels were 6.3 fold lower (Additional file 1: Fig. S2B). No increase in the numbers of cells expressing GFP was observed after day 14–16, suggesting that by this time the majority of both integrated and unintegrated genomes which were destined to be spontaneously active had done so; therefore, this interval could be useful to study durable latency in the GFP-negative cells. In order to be particularly cautious regarding raltegravir efficacy [42], we added this integrase inhibitor 3 times over the course of the experiment, achieving a reduction from 0.48 integrated genomes per GFP +cell to less than or equal to 0.002 iDNA copies per GFP+ cell, i.e. less than one in 500 GFP+ cells contained an integrated provirus. (Additional file 1: Fig. S2C). In addition, the conformity of the raltegravir and integrase mutant results confirms that reversal of raltegravir activity is not playing a role. To test the effect of virus inoculum size on expression from uDNA, we reduced the virus inoculum eightfold, yielding a peak of 2 % GFP+ cells with the integration competent virus, or increased it twofold, observing little or no alteration in the kinetics of virus expression or the relative expression from the No RAL vs. +RAL infections (Additional file 1: Fig. S3A). Important for subsequent latency studies in GFP-negative cells, little HIV-1 RNA was expressed (Additional file 1: Fig. S2B). As mentioned above, we recently reported that common gamma chain cytokines enhance in vitro infection of peripheral blood resting T cell primarily by allowing them to survive early reverse transcription- and Vpr-induced apoptosis rather than through enhancement of virus replication per se [36]. Here and in prior work [6] we found that GFP+ cells were almost exclusively found among the cells which did not proliferate. GFP+ cells showed identical cell cycle status as demonstrated by staining for pyronin Y/7AAD (not shown and [36]). CD69 is increased in GFP+ No RAL cells as we previously demonstrated, secondary to HIV-induced suppression of Foxo1 activity [43].

Next, we stimulated the entire cell population consisting of both GFP+ cells that are productively infected as well as the GFP-negative cells that comprise both uninfected and non-productively infected cells (Fig. 1a–c). αCD3/CD28 beads induce T cell activation, while the combination of a PKC activator prostratin (Pro) and a histone deacetylase inhibitor trichostatin A (TSA) more directly activates virus transcription. Integration-competent HIV-1 (Int-WT, No RAL) responded strongly to each stimulus beginning 1 day after infection (assayed on day 3). However, induction of maximal uDNA expression was only achieved with stimulation from day 7 (assayed on day 9) onward, demonstrating the uDNA requires more time than iDNA to establish responsiveness to these virus stimuli. There was a stimulation-induced increase in the GFP fluorescence of pre-existing GFP+ cells for both No RAL and +RAL cultures. However, stimulation increased the number of GFP+ cells only in the +RAL cultures, while the No RAL cultures remained at similar percentages of GFP+ cells. As we will describe in Fig. 5, there is rapid death of GFP+ cells only in No RAL cultures and this is accelerated by further stimulation. As a result, stimulation-induced cell loss masks latency reversal in No RAL cultures here, necessitating removal of pre-existing GFP+ cells to specifically examine latency reversal, which is specifically explored in the following figures. Equivalent results were obtained using the Int-D116 N mutant (Additional file 1: Fig. S1A). Thus, not only does uDNA display slower expression kinetics, its availability for transactivation is delayed compared with integrating HIV-1 and indicates qualitative differences in gene regulation between iDNA and uDNA.

Importantly, two distinct peaks of GFP expression appeared in the No RAL cultures treated with Pro/TSA days 5–7 (assay days 7–9, arrows in Fig. 1b). PCR analysis in our previous study indicated that the dimmer peak corresponds primarily to expression from unintegrated genomes, while cells in the brighter peak contain at least one integrated provirus [6]. Consistent with this, the bright population was lost upon raltegravir treatment (Fig. 1d) or Int-D116N mutation (Additional file 1: Fig S1B). Pro/TSA increased HIV-1 expression with little change in cell phenotype, including size and scatter profile (Fig. 1d). As a result, these cells remained largely homogeneous, and variations in GFP expression were easily discerned. By contrast, αCD3/CD28 beads generated a high degree of cell heterogeneity, which we hypothesized to have masked these subpopulations in the histograms of Fig. 1b. In fact, these two subpopulations were revealed in the activated T cells when the side scatter (which reports cell heterogeneity) and GFP expression were plotted together (Fig. 1d, Additional file 1: Fig S1B). Therefore, separate and distinct populations expressing HIV-1 from both integrated and unintegrated templates is a common phenomenon when resting CD4 T cells are stimulated several days after infection.

## Response of latent unintegrated vs. integrated HIV-1 to a series of latency reversing agents

**Fig. 2:**
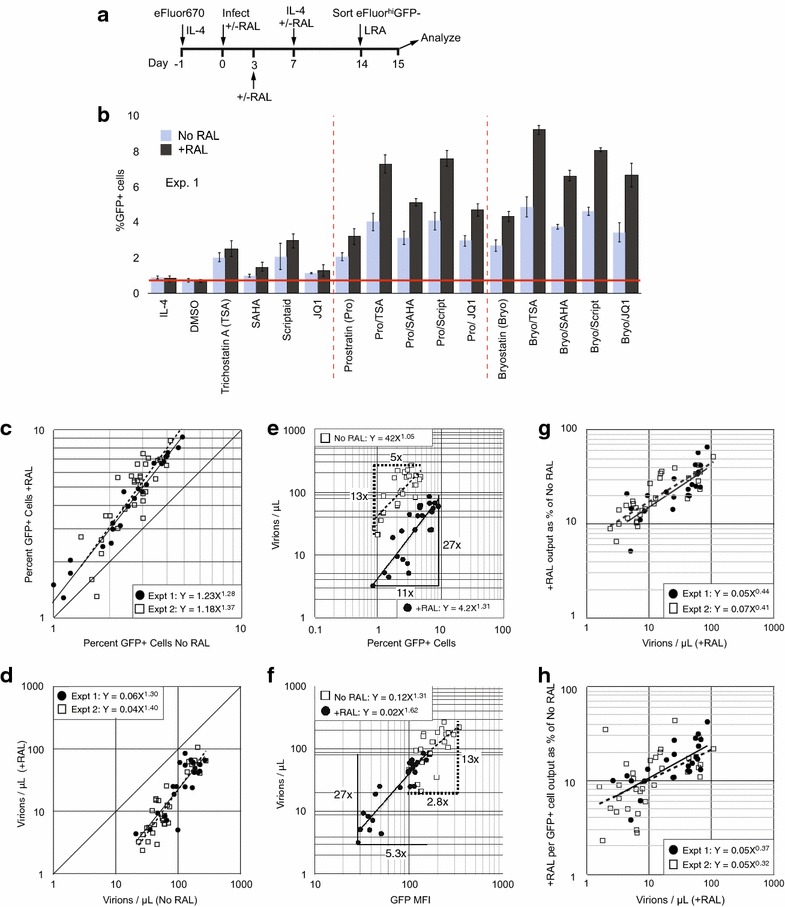
Activation of latent integration-competent HIV-1 and unintegrated HIV-1 with a panel of latency reversing agents (LRA). **a** Experimental design. Raltegravir was added on days 0, 3 and 7 and not removed until sorting. **b** The responses of latently infected cells to various LRAs under No RAL and +RAL conditions, expressed as the percentage of GFP+ cells 1 day after stimulation. Data from one experiment representative of >3 independent experiments with cells from different donors is shown. Each condition was tested in triplicate. Additional file 1: Figure S5 shows an independent experiment (Experiment 2 in Fig. 3c, d, g, h) employing an expanded panel of LRA. Similar results were obtained with the Integrase D116N mutant (not shown). **c** The percentage of GFP+ cells generated in the No RAL vs. the +RAL cultures for each LRA in two independent experiments from **b** (Expt. 1) and in Additional file 1: Fig. S5 (Expt. 2). Each *symbol* represents one LRA from Fig. 2b (Experiment 1) and S5 (Experiment 2). **d** Virus production from No RAL vs. +RAL cultures for both experiments. **e** The percentage of GFP+ cells generated by various LRAs vs. virus release into the culture medium to compare virus production per GFP+ cell. The dynamic range is shown for the number of GFP+ cells and for virus production as fold induction of the maximum over the minimum value. Data are from Expt. 1 and is representative of 3 additional independent experiments. **f** GFP Mean fluorescence intensity (MFI) of the GFP+ cells vs. virus production for No RAL and +RAL cells in Expt. 1. Similar results were obtained from Expt. 2 (not shown). The dynamic ranges are shown as in **e**. **g** Relationship between the strength of the LRA in inducing virus production (X axis) and the +RAL output expressed as a percent of the No RAL output. +RAL output reached 66 % of No RAL output for Bryostatin+ SAHA. **h** Relationship between the strength of the LRA in inducing virus production (X axis) and the +RAL output per GFP+ cell expressed as a percent of the No RAL output per GFP+ cell. **c**–**h** All p ≤ 0.001

We next examined latent uDNA responses to several latency reversing agents (LRA) being considered as components of curative therapies. To this end, we established 14-day infections with the single round GFP reporter virus in the presence or absence of maximally effective raltegravir (Fig. 2a) and sorted eFluor670^hi^GFP-negative cells. Maintenance of the resting CD4 T state after sorting was evidenced by lack of expression of activation markers and G0-G1a cell cycle status in >99.3 % of eFluor670^hi^GFP-negative cells (Additional file 1: Figure S4). We then applied a panel of LRA or combinations of LRA, including PKC activators, HDAC inhibitors and P-TEFb agonists. We measured GFP fluorescence and virus release 24 h after stimulation (Fig. 2b). All LRA which were effective on integration-competent HIV-1 were also effective in reversing latency from uDNA. Prior studies, including our own, have found that unintegrated retroviral genomes are responsive to histone deacetylase inhibitors [6, 44–46], which we confirm. Similar results were obtained in multiple experiments with either raltegravir (Additional file 1: Fig. S5) or with the Int-D116N virus (not shown). Infection without spinoculation resulted in a reduced infection frequency but the responses of latently infected cells to LRA were similar to spinoculated cells (not shown). Sorted eFluor670^low^GFP-negative cells, the proliferated population, did not generate GFP+ cells either spontaneously or following addition of latency reversing agents (not shown). This is interesting because homeostatic proliferation does not substantially alter the latent reservoir in vitro [47], thus neither productive infection nor latency in our system were efficiently established in cells which subsequently proliferated.

**Fig. 3:**
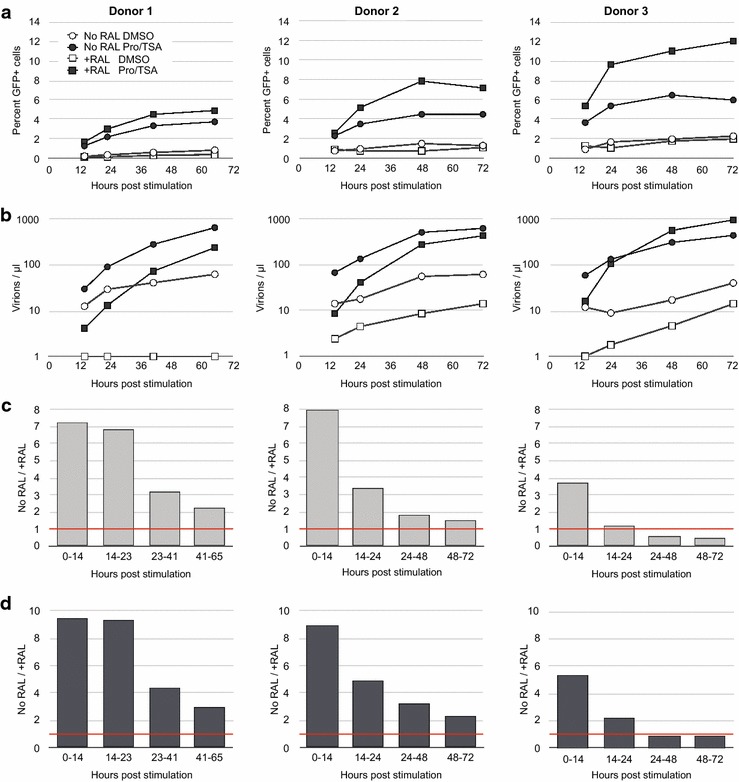
Kinetics of latency reversal and virus production from sorted GFP-negative cells following in vitro infection of resting CD4 T cells from 3 donors. The 14-day latency protocol from Fig. 2a was followed using cells from 3 HIV-negative donors following infection with equal amounts of HIV-1 in order to investigate kinetics of latent virus activation and donor variability. For added precaution against de novo infection, Indinavir was added at day 0 and 7 p.i., while efavirenz was added at day 5 p.i. for donors 2 and 3. **a** Percent GFP+ cells after Pro/TSA or DMSO carrier control treatment of sorted GFP-negative cells. Percent GFP+ cells prior to sort (No RAL, +RAL): Donor 1: 4.9 %, 3.3 %, Donor 2: 16.9 %, 22.4 %, Donor 3: 21.4 %, 17.3 %. **b** Virions released into culture medium measured by RT-qPCR for genomic viral RNA. Lower limit of quantitation was 1 virion/µl. **c** The difference in virus output from the No RAL and +RAL cultures decreases over time following latency reversal. Graphs present the ratio of No RAL virus output vs. +RAL virus output over the indicated interval. Output over each interval for No RAL and +RAL cells was calculated by subtracting the virions/µL at the earlier indicated time from the later time, then the ratio of No RAL/+RAL was graphed. For example, for Donor 1 during the first 14 h (“0–14”) the No RAL cells released 7.3 times as many virions as the +RAL cultures but over the 41–65 h interval, the No RAL cells release 2.2 times as many virions as the +RAL cells. *Red line* indicates equal output from the No RAL and +RAL cells. **d** The difference in virus output per GFP+ cell from the No RAL and +RAL cultures decreases over time following latency reversal. The ratio of No RAL virus output per GFP+ cell vs. +RAL virus output per GFP+ cell over the indicated interval. Output during each interval, calculated as in 3C was then divided by the percent GFP+ cells at the end of each interval. For example, for Donor 1 during the first 14 h (0–14) each GFP cell in the No RAL infections released 9.5 times as many virions as the +RAL GFP+ cells but during 41–65 h interval, the No RAL cells released 2.9 times as many virions per GFP+ cell. Red line indicates equal output from the No RAL and +RAL cells

Strikingly, but also consistent with Fig. 1, at least as many and usually more GFP+ cells were generated from the +RAL infections for each LRA than from the No RAL infections (Fig. 2b, c). This held true for infections at lower and higher MOI (Additional file 1: Fig. S3B) and for Int-D116 N viruses (not shown). An expanded panel of LRA produced similar results (Additional file 1: Fig. S5, and Expt. 2 in Fig. 3c, d, g, f). Fewer virions were generated from the +RAL cells in each culture (Fig. 2d), and fewer virions were released per GFP+ cell (Fig. 2h) that was consistent with the lower transcription from unintegrated genomes. Virus production was highly correlated with GFP fluorescence intensity similarly for the No RAL and +RAL infections (Fig. 2f). However, the dynamic range of the induction of both GFP+ cells and the GFP fluorescence intensities were greater for the +RAL infections (bracketing lines in Fig. 2e, f). This translated into the finding of Fig. 2g, where, as the strength of the activators increased (more virions were released), the +RAL virus production approached closer to the No RAL virus production (Fig. 2g). This increase in the relative output from the +RAL infections was the result of both more GFP+ cells being generated as well as an increase in the output per cell relative to the No RAL cells (Fig. 2h), consistent with the greater dynamic range seen in the GFP fluorescence (Fig. 2f). These results further indicate that uDNA latency and transactivation are regulated differently from integrated proviruses.

With regard to individual LRA and classes of LRA, the PKC agonists (prostratin, bryostatin) were overall the most effective, activating viruses within the most cells and inducing the most virus production from both integrating and unintegrated HIV-1 (Fig. 2b, Additional file 1: Fig S5). Histone deacetylase inhibitors [TSA, SAHA (vorinostat), Scriptaid] and the P-TEFb agonists JQ1 and HMBA only weakly activated latent integrated or unintegrated viruses on their own, but they enhanced virus activation in combination with PKC activators, consistent with the possible enhancement of stochastic fluctuations in transcription [48]. Disulfiram was ineffective in our system on either integrating or unintegrated HIV-1, consistent with other recent reports [49, 50]. Interestingly, synergy between agents was obtained only with PKC activators plus HDACi or JQ1 and only in the raltegravir cultures (Table 1). This provides further evidence that uDNA and iDNA are differentially regulated at the transcriptional level.

**Table 1:** Synergy indices for combinations of latency reversing agents

	No RAL	+RAL
Bryostatin		
+TSA	1.06 ± 0.13	*1.49* *±* *0.28*
+SAHA	0.94 ± 0.07	*1.09* *±* *0.05*
+Scriptaid	0.95 ± 0.05	*1.19* *±* *0.09*
+JQ1	0.85 ± 0.06	*1.14* *±* *0.10*
+HMBA	0.92 ± 0.13	1.12 ± 0.16
+Disulfiram	0.83 ± 0.06	0.79 ± 0.03
Prostratin		
+TSA	1.02 ± 0.04	*1.44* *±* *0.14*
+SAHA	0.96 ± 0.08	*1.30* *±* *0.19*
+Scriptaid	0.97 ± 0.05	*1.42* *±* *0.18*
+JQ1	0.87 ± 0.07	*1.19* *±* *0.16*
+HMBA	0.63 ± 0.10	0.77 ± 0.29
+Disulfiram	0.55 ± 0.14	0.68 ± 0.14
JQ1		
+TSA	0.72 ± 0.08	0.88 ± 0.22
+SAHA	0.87 ± 0.11	0.90 ± 0.11
+Scriptaid	0.84 ± 0.15	0.90 ± 0.10
HMBA		
+TSA	0.63 ± 0.17	0.78 ± 0.03
+SAHA	0.62 ± 0.21	0.81 ± 0.11
+Scriptaid	0.69 ± 0.14	0.91 ± 0.14
Disulfiram		
+TSA	0.68 ± 0.11	0.72 ± 0.13
+SAHA	0.63 ± 0.06	0.69 ± 0.06
+Scriptaid	0.85 ± 0.17	0.98 ± 0.19

Synergy indices were calculated from 3 independent experiments with different cell donors, based on the number of GFP+ cells. Synergy was calculated according to [95] and synergy indices significantly >1 are indicated in italicized text. Additional file 1: Figure S5 contributes data for one of the 3 experiments

A recent study indicated that HIV-1 constructs rendered incapable of replicating by mutations or deletions of the *env* gene can revert to wild type through recombination with envelope expression plasmids following co-transfection of producer cells [51]. To test if replication competent viruses might be contributing to our results, we treated infected resting CD4 T cells with the protease inhibitor indinavir on the day of infection and on day 5 post infection with the non-nucleoside reverse transcriptase inhibitor efavirenz in order to block the spread of any reverted viruses. There was no effect on the generation of latently infected cells, indicating that reversion was not contributing to results (not shown).

## Kinetics of latency reversal

Twenty-four hours after stimulation of latently infected cells is a convenient and frequently utilized time at which to analyze latency reversal [30]. However, our data indicating differential regulation of uDNA and iDNA prompted us to test if uDNA also presents differential kinetics of latency reversal. To this end, we performed a latency experiment with cells from 3 donors, sorting eFluorhiGFP- cells 14 days after infection then stimulating them with Pro/TSA. We analyzed the emergence of GFP expression and virus production over the following 3 days (Fig. 3). During the initial 14 h the No RAL cells generated from 3.7 to 7.9 fold more virus than the +RAL cells (Fig. 3b, c). However, after this initial burst of production, the relative differences in virus output decreased (Fig. 3c). With Donor 3 cells, which had the highest initial infection frequency, the +RAL virus production even exceeded the No RAL production by 2 days after stimulation. We then calculated virus output per GFP+ cell, accounting for the differences in the number of GFP+ cells and changes in their numbers over time (Fig. 3d). On a per-cell basis the rate of virus production from the No RAL cells decreased relative to the +RAL cells. For Donor 3 cells, the output per GFP+ cell equaled the No RAL cells after 2 days. Thus, uDNA latency reversal initially displayed slower kinetics compared with integrated proviruses, but the differences in virus production between the No RAL and +RAL declined over time, indicating a more sustainable virus release from uDNA.

## Distribution of integrated and unintegrated genomes in productive infection and latency

**Fig. 4:**
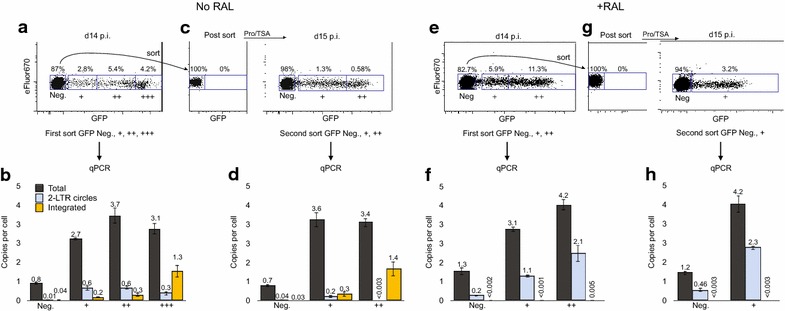
Distribution of integrated and unintegrated HIV-1 DNA after infection resting CD4 T cells and following latency reversal. Data are representative of 5 experiments (see Additional file 1: Table S1 for all experiments). Mean of triplicate PCR and SD are shown. *ND* none detected. **a** At Day 14 p.i., eFluor^hi^ cells from No RAL cultures were sorted based on 4 levels of GFP fluorescence (*blue boxes* indicate sorting gates). **b** Sorted cells were analyzed by qPCR for total HIV-1 DNA, 2-LTR circles and integrated proviruses. **c** GFP-negative cells were sorted, then stimulated with Pro/TSA for 1 day, and then sorted a second time based on GFP expression. **d** Sorted cells were analyzed for viral DNA as in **b**. **e**–**h** The procedures of **a**–**e** were performed on raltegravir treated cells

We next analyzed the content of cells for integrated and unintegrated HIV-1 before and after latency reversal. Fourteen days after direct infection of resting CD4 T cells, we sorted cells into 4 groups based on GFP fluorescence intensity (Fig. 4a). Using qPCR we then directly measured total HIV-1 genomes, integrated proviruses and 2-LTR circles (Fig. 4b). The primers to measure total DNA amplify all integrated and unintegrated species of HIV-1 DNA. 2-LTR circles have been observed to be formed as a minor fraction of the unintegrated species, forming about 1/30th of the total uDNA, with 1-LTR circles and linear uDNA constituting the remainder [52, 53]. The overall amount of uDNA is calculated as total DNA minus integrated DNA.

The most obvious result, consistent with prior studies [54, 55], was that uDNA was formed in excess of integrated proviruses. In this infection of resting T cells, uDNA was present on day 14 p.i. at nearly tenfold excess over iDNA (Additional file 1: Table S1) amongst all cells (GFP+ and GFP-neg.). Cells with the highest GFP fluorescence (+++ cells corresponding to the prominent peak in Fig. 1b) contain at least one integrated genome consistent with prior results [6, 14]. By contrast, single + and double ++ cells contained less than one iDNA per cell (0.2 and 0.3 in the + and ++ cells, respectively), thus between 67 and 80 % of these dimmer cells expressed GFP from uDNA only. In 5 similar experiments utilizing 5 different cell donors, a substantial fraction of GFP+ cells (9–62 %) contained only uDNA (Additional file 1: Table S2).

To examine the contribution of unintegrated DNA to latency we treated sorted GFP-negative cells with Pro/TSA (Fig. 4c), then performed a second sort and analyzed the DNA content of GFP-neg. and GFP+ cells (Fig. 4d). Similar to the initial infection, uDNA predominated in the GFP dim cells while there was at least one integrated provirus in the GFP bright cells. Interestingly, uDNA was still the majority population in the bright cells. A prior study in proliferating cells has shown that unintegrated genomes can constitute a similar proportion of transcriptionally active genomes as integrated proviruses [7], however ours is the first study conducted in resting CD4 T cells and to directly parse cell subpopulations and analyze DNA content. There were 20 times more uDNA than iDNA genomes in the GFP-negative cells from the first sort, but only 5.5 times as many in the newly activated GFP+ cells from the second sort (Additional file 1: Table S1) indicating that silent iDNA genomes were more likely to be activated by the Pro/TSA treatment than were silent uDNA genomes, a result consistent with the differential latency regulation. There could also be a higher proportion of defective or otherwise unresponsive uDNA than iDNA genomes. Importantly, no increase in integration resulted from this stimulation 15 days after infection (Fig. 4b vs. d, Additional file 1: Table S1), consistent with the short (1 day) half-life of pre-integration complexes [56].

Consistent with Fig. 1, raltegravir increased the generation of GFP+ cells from the latent pool (3.2 vs. 1.88 %) while inhibiting integration by ≥280 fold (Fig. 4e–h). Raltegravir also increased the appearance of 2-LTR circles by several fold (Fig. 4f), as expected [6, 57, 58]. As in the No RAL cultures, no increase in integration resulted from this stimulation 14 days after infection, in agreement with our prior study utilizing both raltegravir and integrase active site mutants [6], but in contrast to a recent study in which removal of raltegravir 2–3 days after infection allowed integration to proceed [42]. The difference in results from that study to ours is likely to be the considerable differences in the timing between infection and the removal of raltegravir. In the current study we left raltegravir in the culture for the duration of the 14 days prior to sorting and added it 2 additional times on days 3 and 7. The measured quantity of residual integration events in Fig. 4f and h, likely caused by an integrase-independent mechanism, cannot account for the expression of HIV-1 in the raltegravir-treated cultures.

## Persistence of cells expressing HIV-1 from unintegrated genomes

**Fig. 5:**
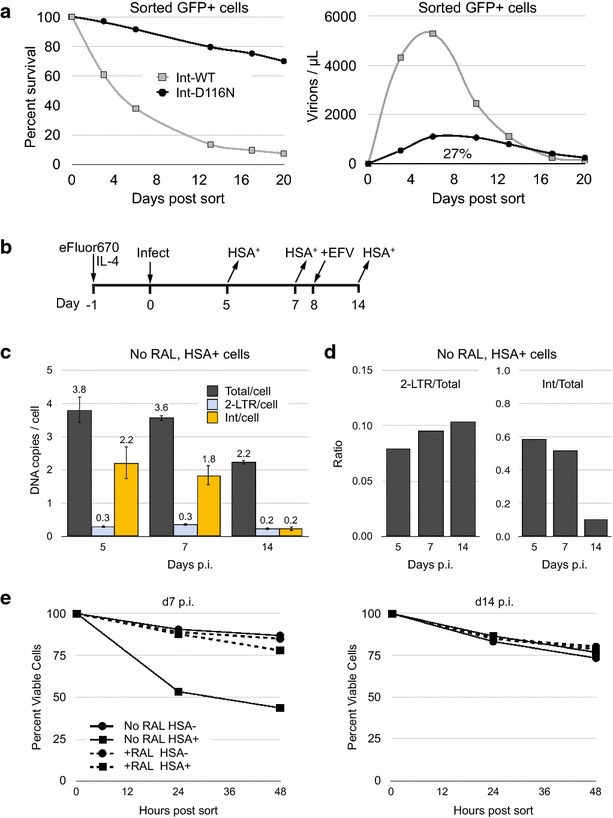
Preferential loss of resting CD4 T cells infected with integrated proviruses. **a** Productively infected GFP+ cells containing Int-WT or Int-D116N HIV-1 were sorted by FACS 7 days after infection and placed back into culture. Survival of the GFP+ cells was assessed by flow cytometry on the indicated days, and virus production from the FACS purified GFP+ cells was measured by RT-qPCR for viral RNA present in the culture medium as described [6]. In this experiment an Envelope+ virus was employed. **b** Experimental design for **c**–**e**. Resting CD4 T cells infected with HSA-reporter viruses were maintained in IL-4 ± RAL for 14 days. At the indicated time points HSA^+^ cells in each sample were isolated using anti-HSA antibody coupled to magnetic beads. Efavirenz was added to all cells on day 8. HSA Positive and negative cells were separated using the MACS system. Cell survival was measured by forward and side scatter analysis. Data present one of two independent experiments. **c** Productively infected HSA^+^ cells purified from No RAL cultures at day 5, 7 and 14 p.i. were analyzed for viral DNA. **d** The ratios of 2-LTR/total HIV-1 DNA in comparison with the ratio of integrated/total HIV-1 DNA for No RAL purified HSA^+^ productively infected cells. **e** HSA^+^ and HSA-neg. cells were purified at day 7 and 14 p.i. and placed into fresh medium. Cell viability of these individual populations was monitored by flow cytometry over 48 h

The lower but more sustained gene expression and de novo virus production from uDNA vs. iDNA suggested the possibility that these are less cytopathic to cells when uDNA is the template. Lower cytopathicity could enhance uDNA persistence and allow more prolonged virus release from the uDNA template. To test this, at day 7 post infection we sorted GFP+ cells infected with an integrase WT HIV-1 or an Integrase active site mutant (Integrase D116N), then analyzed cell survival of the sorted GFP+ cells and virus production from them in the absence of further stimulation (Fig. 5a). As we predicted, the GFP+ cells productively infected with the integrase mutant died at a considerably lower rate than cells productively infected with the integrase WT virus, and they generated 27 % of the total de novo virus output from the Integrase WT virus over this time. After about 12 days in these cells the death curve of the Int-WT virus inflected to be approximately parallel with the Int-D116N virus, suggesting that cells expressing GFP from integrated viruses may have become depleted from the Int-WT infected cells, with relative enrichment of cells expressing GFP from uDNA (shown to arise in Fig. 4) in this non-replicating cell population.

To test this hypothesis, we sampled productively infected cells at 3 intervals after infection and analyzed their DNA content (Fig. 5b–d). Here we employed a reporter virus expressing the cell surface molecule Heat Stable Antigen (HSA, a.k.a. murine CD24) in place of GFP [43] which allows purification of productively infected (HSA+) cells with anti-HSA magnetic beads. Between days 5 and day 7 there was no loss of either integrated or unintegrated HIV-1 DNA in these cells infected without raltegravir added (Fig. 5c, d). However, between days 7 and 14 there was a ninefold reduction in the integrated proviruses among the HSA+ cells but only a 2.6 fold reduction in total DNA at day 7 p.i., consistent with our hypothesis that the cells expressing virus solely from the uDNA template have a survival advantage over cells expressing virus from an integrated template. Confirming our model, HSA+ cells collected on day 7 from the No RAL condition died at a greater rate than either the HSA− cells or HSA+ cells from raltegravir treated culture (Fig. 5e). Cells expressing HIV-1 from uDNA (+RAL, HSA+ cells) died at a similar rate to the HSA-negative cells, confirming that uDNA gene expression is not particularly toxic. All of the cells from day 14 died at an equal rate, which is consistent with the observed depletion of cells expressing HIV from iDNA by this point. In separate experiments, αCD3/CD28 beads accelerated death of purified No RAL HSA+ (or GFP+) cells but not +RAL HSA+ (or GFP+) cells (Additional file 1: Fig. S6). Thus, owing to its reduced toxicity, genetically active uDNA can have a persistence advantage in resting, non-proliferating T cells compared with active integrated proviruses.

## One or two rounds of stimulation do not activate all unintegrated latent genomes

**Fig. 6:**
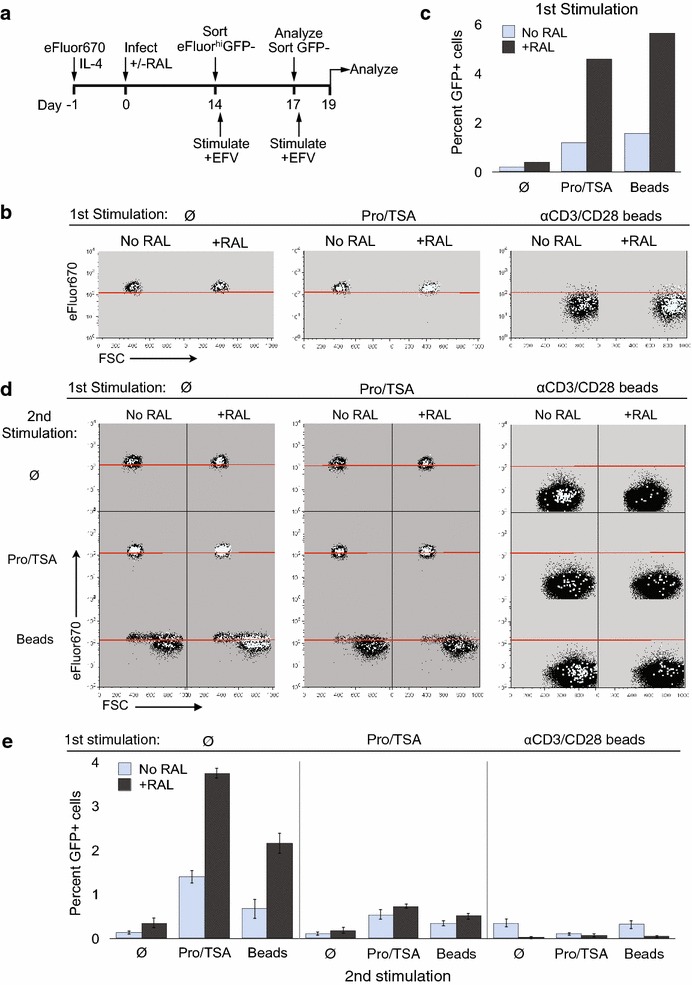
A single round of maximum T cell activation does not induce the expression of all latent unintegrated or integrated viruses. **a** Experimental design. eFluor^hi^GFP-negative cells were sorted on day 14 p.i., then cells were stimulated with Pro/TSA, αCD3/CD28 beads or medium only control (Ø). Three days later (day 17) all GFP-negative cells were purified in a second sort and 30,000 cells per well were (re)stimulated in triplicate. Efavirenz was added on days 14 and 17 to sorted cells as an added precaution against virus spread. **b** Flow cytometric analysis of cells at the end of the first three day treatment. GFP^+^ cells (*light grey dots*) are overlaid on GFP-negative cells (*black dots*). Undivided cells lie above the red lines. **c** The percentage of GFP^+^ cells generated after the first round of stimulation calculated based on the total number of GFP+ of the cells placed in the wells on day 14. **d** Flow analysis of cells after the restimulation. Cells that remained GFP-negative after the first round of stimulation were sorted and equal numbers of these cells were re-stimulated with Pro/TSA, αCD3/CD28beads or medium alone (Ø). **e** The percentage of GFP^+^ cells generated after the second round of stimulation calculated based on the total number of GFP+ of the cells placed in the wells on day 17. Mean and SD for each condition are shown

The efficacy of any shock and kill regimen will require that essentially all replication competent HIV–1 genomes be activated. Prior studies, however, have demonstrated failure of single or repeated strong stimuli to accomplish this in ex vivo and in vitro systems [59, 60]. Here, we investigated whether a single round of strong stimulation can activate all unintegrated latent genomes (Fig. 6). Fourteen days after infection, we treated sorted eFluor^hi^GFP-neg. Cells with Pro/TSA, or activated them with αCD3/CD28 beads plus IL-2, or left them untreated (First Stimulation) (Fig. 6b, c). The αCD3/CD28 beads induced cell enlargement and proliferation in ≥99 % of the cells, indicating essentially complete activation. Pro/TSA, which does not induce T cell activation, resulted in no cell enlargement or proliferation but similar HIV-1 induction. We counted the total number of GFP+ cells generated from the GFP-negative sorted cells, and calculated the percent GFP+ cells based on the number of cells placed in each well before the first stimulation (Fig. 6c). Three days later we sorted GFP-neg. cells a second time from each culture and (re)stimulated them with one of the agents or with nothing (Second Stimulation). This yielded 9 combinations of first and second stimuli (Fig. 6d). We calculated the percent GFP+ cells based on the number of cells placed in each well before the second stimulation (Fig. 6e). Stimulation in the first round reduced the number of GFP+ cells generated in the second round. However, a second stimulation nevertheless generated measurable numbers of GFP+ cells. These finding held true in both the raltegravir treated and untreated cultures. In fact, more GFP+ cells were generated in the raltegravir cultures in the first and second round stimulations, with the exception of cells that were activated by αCD3/CD28 beads in the first round. We previously demonstrated [6] that cell proliferation antagonizes uDNA gene expression, so it seems likely that that this effect plus the partitioning of uDNA genomes as the cells divided reduced the number of uDNA genomes that could be activated in αCD3/CD28 activated cells.

In a variant of this experiment, we stimulated cells infected with the single round virus twice (day 8 and day 11 post infection) prior to sorting for GFP-negative cells. Similar to Fig. 6, we observed latent viruses emerge 2 days later with or without further stimulation, further confirming a strong stochastic component to latency reversal (Additional file 1: Fig. S7). Overall, these results indicate that both uDNA latency and integrated proviral latency can resist a single or even double exposure to stimuli intended to reverse the latent state.

## Failure to down modulate HLA Class I expression and CTL responses to HIV-1 epitopes expressed from integration competent and defective HIV-1

**Fig. 7:**
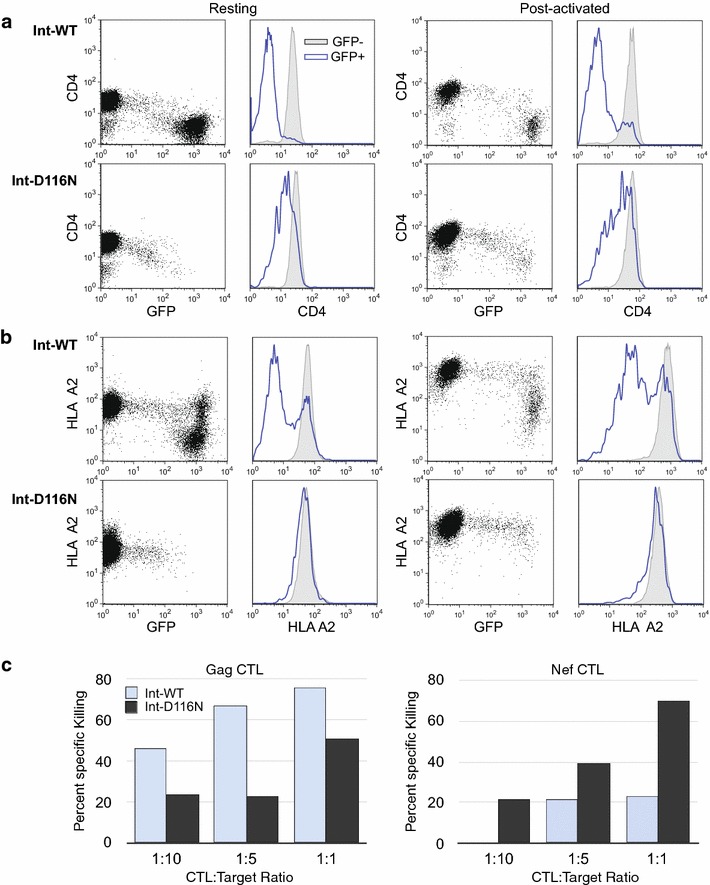
Gene expression from uDNA fails to down modulate HLA Class I but is efficiently recognized by HIV-1-specific CTL. **a** Unintegrated HIV-1 down modulates CD4 in both resting (*left*) and activated (*right*) CD4 T cells. Resting IL-4 treated CD4 T cells were infected then analyzed 6 days after infection with Integrase wild type (Int-WT) or Integrase mutant (Int-D116N) HIV-1 GFP reporter viruses (*left*). One half of the cells were then activated with αCD3/CD28 beads then analyzed 2 days later (*right*). Activation caused a loss of a portion of GFP+ cells, resulting in fewer GFP+ at the time of analysis. The positive control CD4 down modulation data for the integrase WT virus was also a positive control in [43]. **b** Only integration-competent HIV-1 down modulates HLA A2 in resting (*left*) and post-infection activated (*right*) CD4 T cells. Cells were treated as in A but stained with an anti-HLA A2 antibody. **c** CD8+ cytotoxic T cells that recognize defined Gag and Nef epitopes (see materials and methods) specifically kill GFP+ cells infected with Int-WT or Int-D116N HIV-1. Killing was measured by loss of GFP+ cells, with non-specific killing of HLA Class I mismatched controls subtracted from the total. One of two representative experiments is shown

Observations of gene expression from uDNA prompted the notion that this could expand the antigenic repertoire of HIV-1 and the number of cells vulnerable to CTL recognition [61]. We first measured the ability of uDNA to down modulate receptors required for immune recognition. In our system, integration-competent HIV-1 efficiently down modulated CD4 (Fig. 7a) and HLA Class I (Fig. 7b) in the majority of either resting CD4 T cells or cells subsequently activated according to the post-infection activation (Post-activation) procedures utilized in this work and in our prior study [6] which demonstrated that high level uDNA expression is only achieved by this sequence of events and not by infection of pre-activated T cells. Post-activation of the cells resulted in the death of more GFP+ cells than GFP-neg. cells, resulting in a decrease in the percent GFP+. HLA Class I down modulation by the integrating virus was biphasic and observed only in cells with GFP expression higher than achieved by uDNA. HIV-1 Nef-dependent HLA Class I down modulation (reviewed in [62]) has been shown to inhibit CTL recognition [63]. Similar to previous reports [64] uDNA was able to down modulate CD4, though not as efficiently as integrating virus. On the other hand, HLA Class I was barely affected by uDNA gene expression, suggesting continued high vulnerability to CTL recognition might result.

Next, we examined the ability of HIV-1-specific CTL to recognize and eliminate cells expressing HIV-1 proteins from unintegrated templates (Fig. 7c). CD8 cytotoxic T cell lines specific for HIV-1 Nef and Gag were developed from HIV-1 infected individuals as previously described [65, 66]. These CTL lines were co-cultured with HIV-1-infected HLA Class I-matched target cells at varying CTL to target cell ratios. Negative controls included HLA Class I mismatched infected target cells. Killing of HIV-1 expressing cells was measured as a reduction in the percentage of GFP+ cells by flow cytometry, and non-specific killing of the HLA Class I mismatched cells was subtracted from these data. Both Gag-specific and Nef-specific CTL recognized and eliminated GFP+ cells expressing HIV-1 proteins from HIV-1 with wild-type integrase or from uDNA (Int-D116N) with the efficiency of killing titrating according to the CTL:target ratio. Perhaps not-surprisingly, given the weaker expression of Gag vs. Nef from uDNA [6], CTL against Gag more efficiently killed Int-WT infected cells than Int-D116N infected cells. This would be consistent with a recent study indicating that 2-LTR uDNA is cleared less efficiently by CTL than integrated proviruses [67]. On the other hand, Nef CTL more efficiently killed cells infected with the integrase deficient virus, so uDNA clearance could be epitope-specific. It is tempting to speculate that failure to down modulate HLA Class I could allow greater vulnerability to CTL killing of cells expressing early vs. late gene products from unintegrated HIV-1.

## Discussion

The findings presented here and in our prior studies [6, 14, 68] challenge the notion that uDNA is inevitably a replicative dead end. Instead, latency, gene expression and virus production from unintegrated HIV-1 DNA are natural and possibly unavoidable consequences of the direct infection of resting CD4 T cells. In this in vitro setting, we found evidence that gene expression from uDNA is regulated somewhat differently from iDNA during productive infection, as only uDNA required an interval of several days after infection to obtain responsiveness to activating agents. Furthermore, latent uDNA demonstrated a wider dynamic range of responses to latency reversing agents and slower activation kinetics compared to latent integrated proviruses. Our prior work demonstrated that uDNA but not iDNA depends on virion-associated Vpr for expression in resting T cells further demonstrates qualitative differences in gene regulation between these forms of HIV-1 genomes [6].

HIV-1 uDNA surprisingly had a persistence advantage over integrated proviruses in resting CD4 T cells, which we attribute to reduced cytotoxicity resulting from uDNA’s lower gene expression. As a result, resting CD4 T cells expressed proteins and viruses from unintegrated genomes for longer than from integrated proviruses. The extended persistent expression from uDNA vs. iDNA was evident both during initial infection of cells and after reversal of latency. We also found that uDNA failed to down modulate HLA Class I surface expression on resting CD4 T cells, and HIV-1-specific CTL efficiently killed cells infected with unintegrated HIV-1. Thus, in vivo, uDNA may contribute to the development of anti-HIV-1 immune responses and to immune-dependent cell loss.

The de novo synthesis of virions from unintegrated HIV-1 requires a particular set of conditions that is clearly not the majority circumstance in vivo, and virus production by unintegrated HIV-1 in resting CD4 T cells is usually about one order of magnitude lower than from viruses which were capable of integration. Importantly, in vivo, most viruses are generated from activated T cells, which generate several-fold more viruses than resting T cells [69], and which we confirmed in [6] do not support high level gene expression and de novo virus production from uDNA. Thus, the finding that uDNA is capable of latency and virus production do not challenge the accepted view that HIV-1 predominantly replicates via the successfully integrated provirus. These findings are also consistent with the documented efficacy of raltegravir in vivo. We have identified an exception to the rule regarding the necessity of integration for de novo virus production and persistence. Intriguingly a recent study observed persistence of an integrase-defective HIV-1 in a patient [70], and infectious virus production from uDNA has recently been reported in T cell lines [71].

Investigations into the regulation of HIV-1 latency rely heavily on in vitro models, with several prominent systems utilizing direct infection of resting T cells (reviewed in [30]). In light of the results presented here, the contribution of uDNA to systems employing direct infection of resting and/or non-proliferating cells warrants investigation, as these systems may generate some of their output (RNA, virions, GFP+ cells) from unintegrated HIV-1 DNA, especially when stimulation is provided at least 5 days after infection. In addition, the procedure we established to reveal HIV–1 replication from uDNA [6] parallels the protocols developed to characterize in vivo latency: culture resting CD4 T cells from HIV+ individuals ex vivo for about 6 days, then activate them [56, 72, 73]. This culture period was performed so that pre-integration complexes in recently infected cells, which have a half-life of 1 day [40], would disintegrate and the cDNA would not integrate in response to T cell activation. Our studies suggest that these procedures would in fact optimize gene expression from any remaining unintegrated HIV-1. On the other hand, when infected cells proliferate, uDNA gene expression does not lead to de novo virus production [6], so systems in which activated and dividing T cells are infected are unlikely to generate such high levels of uDNA gene expression [74, 75]. The use of a proliferation tracking dye (eFluor670) allowed us to isolate only the non-proliferated cells for study of the in vitro generated latent reservoir, where we found the vast majority of latent infection. This technique is potentially useful in other models of HIV latency where the cells have been stimulated or activated prior to infection.

The potential of shock and kill therapies to eliminate latent reservoirs will depend upon these cells dying after virus activation, either via viral cytopathic effects or by immune killing [76], which has been cast into some doubt [77]. Our data here indicate that gene expression from uDNA displays slower kinetics and is more benign to host cells than from integrated proviruses, so viral cytopathicity is a particularly unreliable mechanism for eliminating cells expressing HIV-1 from uDNA. The lack of uDNA cytopathicity in resting CD4 T cells counterintuitively translated into the aforementioned persistence of unintegrated vs. integrated genomes in our system. The generation of uDNA may bias against the kill portion of investigations performing direct infection of resting T cells.

Success of shock and kill will also depend upon activating all latent replication competent genomes; however, this task is complicated by the multifactorial nature of HIV-1 latency [78]. Among these factors, the availability and activity of transcription factors in cells of varying lineages and activation states, the installation of epigenetic controls, and virus integration into chromosomal regions that favor or disfavor gene expression can all complicate the development of universally effective LRA [79, 80]. Additionally, cellular activation does not necessarily induce all latent genomes [60, 81], and subpopulations of latently infected cells may display varying “degrees of latency” [82] or are regulated with a strong stochastic component [81, 83]. This variability obtains even to clonal latent cell lines [38]. Here, we provide evidence for a further complication that can apply to, at least, in vitro models of latency: unintegrated HIV-1 genomes contribute to latency when resting CD4 T cells are directly infected, and, as has observed for integrated proviruses, a subset of latent uDNA genomes resist activation by single or double stimulation and contain a strong stochastic component to their expression. Our finding that uDNA gene expression is regulated by somewhat different rules than integrated proviruses could also complicate in vitro testing of therapeutics to purge viral reservoirs.

Two basic and non-exclusive models for the establishment of HIV-1 latency have been proposed. Siliciano and Chun, after analyzing quiescent blood CD4 T cells, initially suggested that a rapid return to quiescence of recently infected activated cells can induce transcriptional silencing, as necessary transcription factors are withdrawn. This model has the logical benefits that (1) most in vivo latency in blood resides within previously activated memory CD4 T cells, and (2) it has been commonly accepted, based on studies of purified blood T cells, that quiescent (resting) CD4 T cells are poorly infectible, though infection and latency are possible even without stimulation [24]. However, the blood contains only 2 % of the body’s lymphocytes, with 98 % resident in tissues. Lymphoid and mucosal tissues are the predominant sites of HIV-1 replication, depletion and latency [29, 84]. Microenvironmental factors in tissues enable resting CD4 T cells to be directly infected by HIV-1, and these cells are the dominant targets of early infection [29]. It has also been shown that infection of both resting cells from lymphoid tissues and activated T cells results in rapid establishment of latency [25]. Thus, a possible second mechanism contributing to HIV-1 latency has been gaining traction, in which direct infection of resting but permissive cells allows HIV-1 to rapidly enter latency immediately or soon after reverse transcription and integration [24]. Within this scenario, the present work demonstrates that latency and ultimately virus replication can be established in the absence of integration.

The responses of latent uDNA to the panel of latency reversing agents, measured as the induction of GFP+ cells and virions from sorted GFP-negative cells treated with raltegravir, obeyed the same basic pattern as when integration was allowed to proceed. However, the dynamic range of de novo virus production was substantially greater from unintegrated HIV-1, with weak stimuli inducing roughly equal numbers of GFP+ cells and 7–20 % as many virions as when integration is not blocked, while strong stimuli induced 2–3 times as many GFP+ cells and 30–66 % as many virions within 24 h and up to >200 % more virions over 3 days. Thus, as the strength of the stimulus increased, relatively more uDNA genomes were recruited out of latency compared with integrated proviruses. This greater dynamic range of uDNA responses to LRA suggests that uDNA transcription is regulated differently from iDNA. Chromatin structures that are proximal to the HIV-1 transcriptional regulatory regions, chief among these being promoter-proximal nucleosomes, regulate the susceptibility of HIV-1 to transcriptional activation. The ability of histone deacetylase inhibitors to reverse latency is thought to be due at least in part to the displacement of promoter-proximal nucleosomes following histone acetylation. The responsiveness of uDNA from HIV-1 and other retroviruses to HDACi, which has been observed before by others and by us [6, 44–46], is an indication that uDNA is also regulated by chromatin [44]. HDACi also activate many cellular genes, so the influences of HDACi on HIV-1 expression are likely complex and include multiple indirect effects. Nevertheless, our conclusion that uDNA is transcriptionally regulated by a set of criteria somewhat different from iDNA is supported by the notion that uDNA would lack chromosomal positional effects that an integrated gene would be subjected to, such as integration sites which favor or disfavor transcription, and integration near transcriptional regulatory sequences of cellular genes. In addition, Vpr’s ability to transactivate uDNA [85], and our prior demonstration that Vpr is the critical determining factor in establishing uDNA but not iDNA transcriptional competence in resting CD4 T cells, emphasizes the differential transcriptional regulation of uDNA and iDNA [6]. Use of Δvpr HIV-1 vectors to infect resting CD4 T cells would fail to achieve expression from unintegrated viral DNA and could account for some of the variability among systems [86]. How Vpr may contribute to latency regulation is not clear, but some prior studies, including those from one of us, have indicated that Vpr can reactivate latent proviruses [87–90]. More recently, the cellular protein family TASK and the viral accessory protein Vpu has been shown to specifically down-modulate HIV-1 uDNA transcription in a NF-κB-depended manner [91].

Antiviral immunity, particularly cytotoxic T cells, is envisioned to assist eradication of cells containing recently activated viruses. However, CTL killing in this scenario cannot necessarily be taken for granted [77, 92], and CTL responses may not be efficient at sites of HIV-1 replication in lymphoid tissues [93]. We observed that uDNA expression is unable to efficiently down modulate HLA Class I expression in resting or CD4 T cells that were subsequently activated, despite being able to down modulate CD4 expression [64]. HLA Class I is targeted by HIV-1 Nef, which has been shown previously to be expressed from uDNA, however, relatively higher levels of Nef are required to induce HLA Class I down modulation than CD4 down modulation. We predicted that failure to down modulate HLA Class I might leave cells expressing HIV-1 from uDNA particularly vulnerable to CTL killing. We observed that both Nef-specific and Gag-specific CTL killed autologous target cells infected either with integrase wild type HIV-1 or integrase defective virus. Interestingly, cells infected with integrase defective virus were killed more efficiently than WT virus by the Nef-specific CLT but less efficiently by the Gag CTL. It is tempting to speculate that this relates to the relative inefficiency of Gag expression by uDNA, especially in replicating cells [6]. Recent studies have shown that the viral reservoir is influenced by the susceptibility of cells to CTL killing [67, 94], and it will be important to examine how uDNA expression sensitizes various cell types at different activation states to these processes.

## Methods

### Viruses

Viruses have been described previously [6, 14, 37, 43]. With the exception of Fig. 5a which employed an Env+ virus, infectious virions were generated by co-transfection of 293T cells with a plasmid expressing the *env* gene-defective reporter virus and a plasmid expressing the CXCR4 trophic HIV-1 NL4-3 envelope, as described previously [6, 14, 37]. Virus stocks were filtered through a 0.45 μm pore-sized filter and treated with benzonase (50 μ/ml, Novagen) to remove residual plasmid remaining from transfections, as described [14].

### Cells and infections

Peripheral blood buffy coats from HIV–1-negative adults were purchased from the New York Blood Center. Isolation, culture, IL–4 treatment, eFluor670 staining and infection of resting CD4 T cells have been described previously [6]. p24gag ELISA analysis on viral stocks indicated a range of 175–320 ng p24gag/10^6^ cells except as indicated in Additional file 1: Fig S3. The titers of the virus stocks were routinely determined by TaqMan RT-qPCR for HIV-1 RNA and normalized as previously described [6]. Raltegravir (1 μM in water) was added immediately after infection and supplemented at day 3 and day 7 after infection. Maximally effective raltegravir dose in resting CD4 T cells was determined in [6] to be 0.3 µM. The HIV-protease inhibitor indinavir (IND) was used at 2 μM in water and the NNRTi efavirenz (EFV) was used at 1 μg/ml in DMSO. All antiretrovirals were obtained from the NIH AIDS Research and Reference Reagent Program.

### Stimulation of cells with latency reversing agents

The following conditions were utilized: αCD3/CD28 T cell activation beads (1 μl/40,000 cells; Life Technologies), PHA-L (3 μg/ml in PBS; Sigma), TNF-α (10 ng/ml in PBS; Invitrogen), PMA (16.2 μM in DMSO; Fisher Scientific), prostratin (Pro; 330 nM in DMSO; Santa Cruz Biotechnology), bryostatin-1 (30 nM in DMSO; Santa Cruz Biotechnology), trichostatin A (TSA; 130 nM in DMSO; Fisher Scientific), JQ1 (1 μM in DMSO; kindly provided by James E. Bradner, Dana Farber Cancer Institute, Boston, MA), disulfiram (50 nM in DMSO; Tocris Bioscience), HMBA (1 mM in dH_2_O; Sigma), valproic acid (5 mM in PBS, Sigma), SAHA (vorinostat; 335 nM in DMSO; Santa Cruz Biotechnology), scriptaid (1.8 μM in DMSO; Santa Cruz Biotechnology), oxamflatin (500 nM in DMSO; Santa Cruz Biotechnology) and DMSO carrier control (0.1 % [14 mM], specific to Pro/TSA; Sigma). In the latency activation experiments, GFP-negative cells were plated at 30,000 cells per well in 96-well plates for stimulation. A supernatant sample from each treatment was collected 24 h later or at the indicated time point and cell GFP expression was concurrently analyzed by flow cytometry. Synergy index was calculated based on published methods [95]. Briefly: The percentage of GFP+ cells after stimulation by each drug was converted to fold induction by normalizing to the percentage of GFP+ cells in IL-4-only treatment. The fold induction of a combination treatment was then divided by the sum of the fold induction of each drug when used separately. A combination with an index of >1 was considered synergistic, while an index of 1 was considered to be additive and <1 was considered as inhibitory.

### Flow cytometry

Flow cytometric analysis was performed on a FACSort flow cytometer (Becton–Dickinson) upgraded to 3 lasers and 5 color channels as previously described [6]. Cell proliferation was calculated using the Proliferation platform of the FlowJo software based on eFluor670 intensity. Anti-CD4 (RPA-T4), Anti-HLA Class I (HLA A2) PerCP-Cy 5.5 (BB7.2), Anti-HLA-DR PerCP-Cy 5.5 (G46-6) and Anti-HSA-PE (M1/69) antibodies were purchased from BD Biosciences. Anti-CD69 PerCP-Cy5.5 (FN50), and isotype control mouse IgG1κ PerCP-Cy5.5 (MOPC-21) antibodies were purchased from BioLegend. Anti-CD25 PerCP-Cy 5.5 (BC96) antibody was from eBioscience. Cell sorting was performed using a BD FACSAria sorter at the NYU Langone Medical Centre Office of Collaborative Science Flow Cytometry Core Facility. Flow cytometry for the CTL killing assay was performed at the UAB Center for AIDS Research Flow Cytometry Core. Intracellular DNA and RNA were stained with 7-aminoactinomycin D (7-AAD) and pyronin Y (PY) as previously described [96].

### MACS separation of productively infected and non-productively infected cells

After infection with murine HSA (CD24)-expressing reporter virus, primary CD4 T cells were treated at 1 day p.i. with pronase (Fisher Scientific) to remove residual HSA molecules from the initial infection as in [14]. At the indicated time points, live cells were purified on a Ficoll-Paque (GE Life Sciences) gradient, stained with a phycoerythrin (PE) labeled anti-mCD24 antibody (M1/69, BD Biosciences) and then bound to anti-PE magnetic microbeads (Miltenyi Biotec). HSA Positive and negative cells were separated using the MACS system (Miltenyi Biotec) according to manufacturer’s protocol. Purity was routinely >95 %. Cell survival was measured by forward and side scatter analysis.

### PCR methods for quantification of HIV-1 DNA and RNA

All methods, including primers and TaqMan probes have been previously described [6, 43]. Briefly, DNA from cells was purified using the DNeasy Blood & Tissue kit or the AllPrep kit (Qiagen). Cell associated RNA and viral RNA in culture medium were prepared using the RNeasy kit (Qiagen). DNA was analyzed using the QuantiTect Probe PCR kit (Qiagen) and RNA was analyzed by RT-qPCR using the QuantiTect Probe RT-PCR kit (Qiagen). Integrated HIV-1 was detect by Alu PCR as described [6].

### CTL cell killing assay

HLA I-restricted CD8^+^ Cytotoxic T cell lines reactive against HIV-1 peptides were developed from HIV-1+ individuals as described [97]. CTL clone 2B6 recognizes HLA B*5301 restricted Gagp24-QW9 (QASQEVKNW) epitope in Nef, while clone 2A3 recognizes HLA B*4002 restricted Nef-KL9 (KEKGGLEGL) epitope. Both these clones were obtained from a chronically HIV clade B infected patient expressing HLA-A*02(01, 09)/A*3201; B*4002/B*5301 and Cw*0202/Cw*0401. EBV transformed B lymphoblastoid cell-lines (BLCL) were developed from this patient’s PBMC for use as targets in the killing assay. An HLA-I mismatched BLCL was used as a negative control (HLA-A*2904/A*3002; B*1503/B*5802; Cw*0202/Cw*0602). Target cells were infected with VSV-G pseudotyped NLENG1-ES-IRES or NLENG1-ES-IRES-D116N as described [14, 37], resulting in 5–20 % GFP+ cells. Two days after infection, 50,000 infected target cells were co-cultured for 4 h with the CTL a three CTL:target ratios indicated in Fig. 7c. Cells were then harvested and stained with anti-CD19-PE-Cy7, anti-CD3-Pacific Blue, and anti-CD8-PerCP-Cy5.5 (BD Biosciences). Flow cytometry was performed on a BD FACSAria at the University of Alabama at Birmingham Center for AIDS Research Flow Cytometry Core Facility. Live cells were identified by side scatter and forward scatter profile, and live CD19+ CD3−CD8− cells were analyzed for percent GFP expression. Specific CTL killing of GFP+ target cells was calculated in two steps: The GFP+ cells remaining after CTL exposure was calculated as a percentage of the number of CD19+ CD3−CD8−GFP+ cells present without CTL, then non-specific killing of HLA-mismatched target cells (<10 % in all instances) was then subtracted from this number for each CTL:target ratio. This number was graphed as the Percent Specific Killing in Fig. 7c.

## Additional file

10.1186/s12977-015-0234-9 **Figure S1** (supplement to Fig. 1). Integrase D116N mutant expression in resting CD4 T cells. An experiment performed independently from those in Figs. 1 and 2 was performed using a different donor. (A) Kinetics of virus expression with and without stimulation. (B) Loss of GFPhi  population similar to raltegravir treatment of WT virus.    **Figure S2** (supplement to Fig. 1). Protein, RNA and DNA in GFP+ and GFP- cells at day 14 post infection. (A) HIV-GFP expression in cells at day 14 for analysis in E. Data are representative of >5 independent experiments using cells from different donors. MFI = mean fluorescence intensity of GFP. (B) Intracellular HIV-1 RNA in sorted cells from integration permissive (No RAL) and integration inhibited (+RAL) cultures. Early Rev-independent Fully spliced RNA, and late Rev-dependent unspliced RNA were quantified by RT-qPCR (see methods). Data are normalized to Fully spliced RNA in the GFP+ No RAL cells set as 100%. Data from one of two similar experiments is shown. Standard deviations are from PCR triplicates. Non-proliferated cells were sorted for this analysis (Fig S4A-B). (C) qPCR analysis of Total and Integrated HIV-1 DNA in cells from Fig. 1E-F. Infection in the presence of raltegravir resulted in less than 1 in 500 GFP+ cells containing an integrated provirus.    **Figure S3** (supplement to Fig. 1). Lack of influence of MOI on the timing of HIV-1 gene expression and on No RAL vs. + RAL latency in resting CD4 T cells. (A) Cells were infected as in Figure 1 except that different doses of HIV-1 were applied. Figure 1 employs 300ng p24gag per million cells. Note that the Y axis scales are adjusted to allow comparison of relative kinetics at different MOI. (B) Relative amounts of latency for No RAL and +RAL infections remain constant with varying MOI. GFP-negative cells from each infection were sorted at Day 14 p.i. by FACS and stimulated with Pro/TSA in triplicates or DMSO. Expression of GFP one day after stimulation is shown. Data representative of 3 independent experiments with 3 different donors.    **Figure S4** (supplement to Fig 1). Homeostatic proliferation of IL-4 treated cells and resting status of the non-productively infected cells. (A) eFluor670 and GFP expression on day 14 p.i. Sorting gates are indicated by red boxes. Data are representative of >5 independent experiments using cells from different donors. (B) Analysis of cell proliferation on the day of sorting. The FlowJo Proliferation Platform was used to calculate the percentage of cells that had divided at least once. (C) Expression of T cell activation markers CD69, HLA-DR and CD25 on the eFluorhiGFP-negative cells one day after sorting. Data are representative of three independent experiments. (D) 7AAD-Pyronin Y staining of eFluorhiGFP-negative cells one day after sort. As a positive control for cell cycle progression, uninfected CD4 T cells were activated with αCD3/CD28 beads four days prior to analysis. The arrow with * indicates cells with increased DNA content but low RNA levels that are apparently preparing for homeostatic proliferation. Data are representative of two independent experiments. **Figure S5** (supplement to Fig 2). Expanded panel of latency reversing agents in a donor different from Fig. 2. Grey and black vertical lines represent expression levels from the IL-4-only negative controls for No RAL and +RAL conditions in order to better visualize induction from latency. (A) Percent GFP+ cells as in Fig. 2 for the expanded panel. (B) Mean fluorescence intensity (MFI) of the GFP+ cells. **Table S1** (supplement to Fig 4). Calculations of ratios of uDNA to iDNA in sorted No RAL cells. Calculations of the ratios of integrated to total DNA in sorted cell populations for Fig. 4A and C. **Table S2** (Supplement to Fig 4). Percentage of cells with only uDNA from 4 additional experiments. Results from 4 additional independent experiments utilizing different donor cells for comparison with Fig. 4. **Figure S6** (supplement to Fig 5). Activation of productively infected cells accelerates death only when integration is allowed. HSA+ cells were isolated 8 days after infection and stimulated or not with αCD3/CD28 activation beads. Viability was assessed at the indicated times by flow cytometry as in Fig. 5E. Similar results were obtained using the GFP virus and sorted GFP+ cells (not shown).   **Figure S7 **(supplement to Fig 6). Two rounds of stimulation by Pro/TSA did not reverse all HIV-1 latency. (A) Experimental design. Infected cells were treated with DMSO (B) or Pro/TSA (C) on day 8 p.i., then washed and re-treated with the same compounds on day 11 post infection. GFP-neg. cells were sorted on day 14 p.i. and treated with IL-4, Pro/TSA or αCD3/CD28 activation beads + IL-2 for 2 days before analysis. Indinavir was added at day 0 and 7 p.i. as an additional precaution against second round infection.
