# Supplementary material for: HIV-1 latency and virus production from unintegrated genomes following direct infection of resting CD4 T cells
Source: Retrovirology. 2016 Jan 5;13:1. doi: 10.1186/s12977-015-0234-9 (PMC4700562; doi:10.1186/s12977-015-0234-9)
Supplement: Supplementary file 1 — 10.1186/s12977-015-0234-9 Figure S1 (supplement to Fig. 1). Integrase D116N mutant expression in resting CD4 T cells. An experiment performed independently from those in Figs. 1 and 2 was performed using a different donor. (A) Kinetics of virus expression with and without stimulation. (B) Loss of GFPhi population similar to raltegravir treatment of WT virus. Figure S2 (supplement to Fig. 1). Protein, RNA and DNA in GFP+ and GFP- cells at day 14 post infection. (A) HIV-GFP expression in cells at day 14 for analysis in E. Data are representative of >5 independent experiments using cells from different donors. MFI = mean fluorescence intensity of GFP. (B) Intracellular HIV-1 RNA in sorted cells from integration permissive (No RAL) and integration inhibited (+RAL) cultures. Early Rev-independent Fully spliced RNA, and late Rev-dependent unspliced RNA were quantified by RT-qPCR (see methods). Data are normalized to Fully spliced RNA in the GFP+ No RAL cells set as 100%. Data from one of two similar experiments is shown. Standard deviations are from PCR triplicates. Non-proliferated cells were sorted for this analysis (Fig S4A-B). (C) qPCR analysis of Total and Integrated HIV-1 DNA in cells from Fig. 1E-F. Infection in the presence of raltegravir resulted in less than 1 in 500 GFP+ cells containing an integrated provirus. Figure S3 (supplement to Fig. 1). Lack of influence of MOI on the timing of HIV-1 gene expression and on No RAL vs. + RAL latency in resting CD4 T cells. (A) Cells were infected as in Figure 1 except that different doses of HIV-1 were applied. Figure 1 employs 300ng p24gag per million cells. Note that the Y axis scales are adjusted to allow comparison of relative kinetics at different MOI. (B) Relative amounts of latency for No RAL and +RAL infections remain constant with varying MOI. GFP-negative cells from each infection were sorted at Day 14 p.i. by FACS and stimulated with Pro/TSA in triplicates or DMSO. Expression of GFP one day after stimulation is [file 12977_2015_234_MOESM1_ESM.pdf]

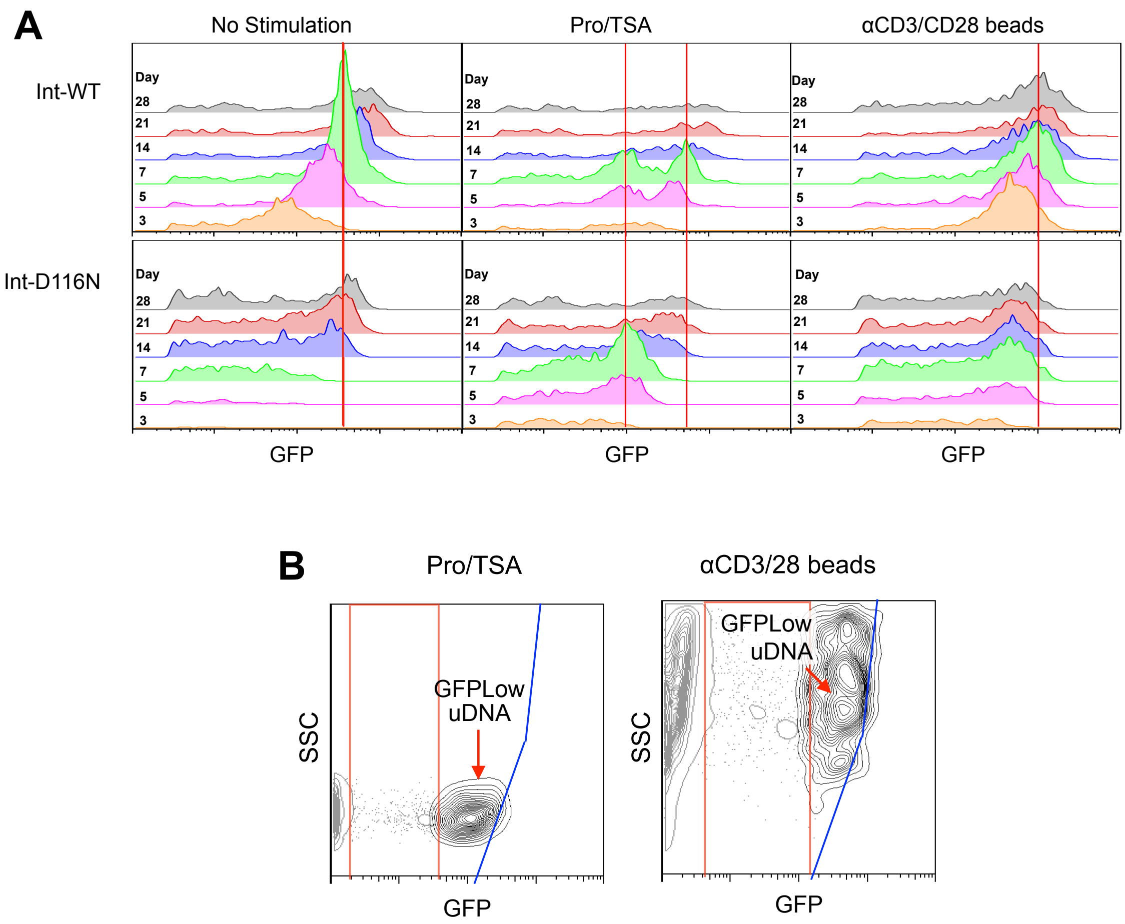

Figure S1 (supplement to Fig. 1). Integrase D116N mutant expression in resting CD4 T cells.
An experiment performed independently from those in Figs. 1 and 2 was performed using a different donor.
(A) Kinetics of virus expression with and without stimulation.
(B) Loss of GFPhi population similar to raltegravir treatment of WT virus.

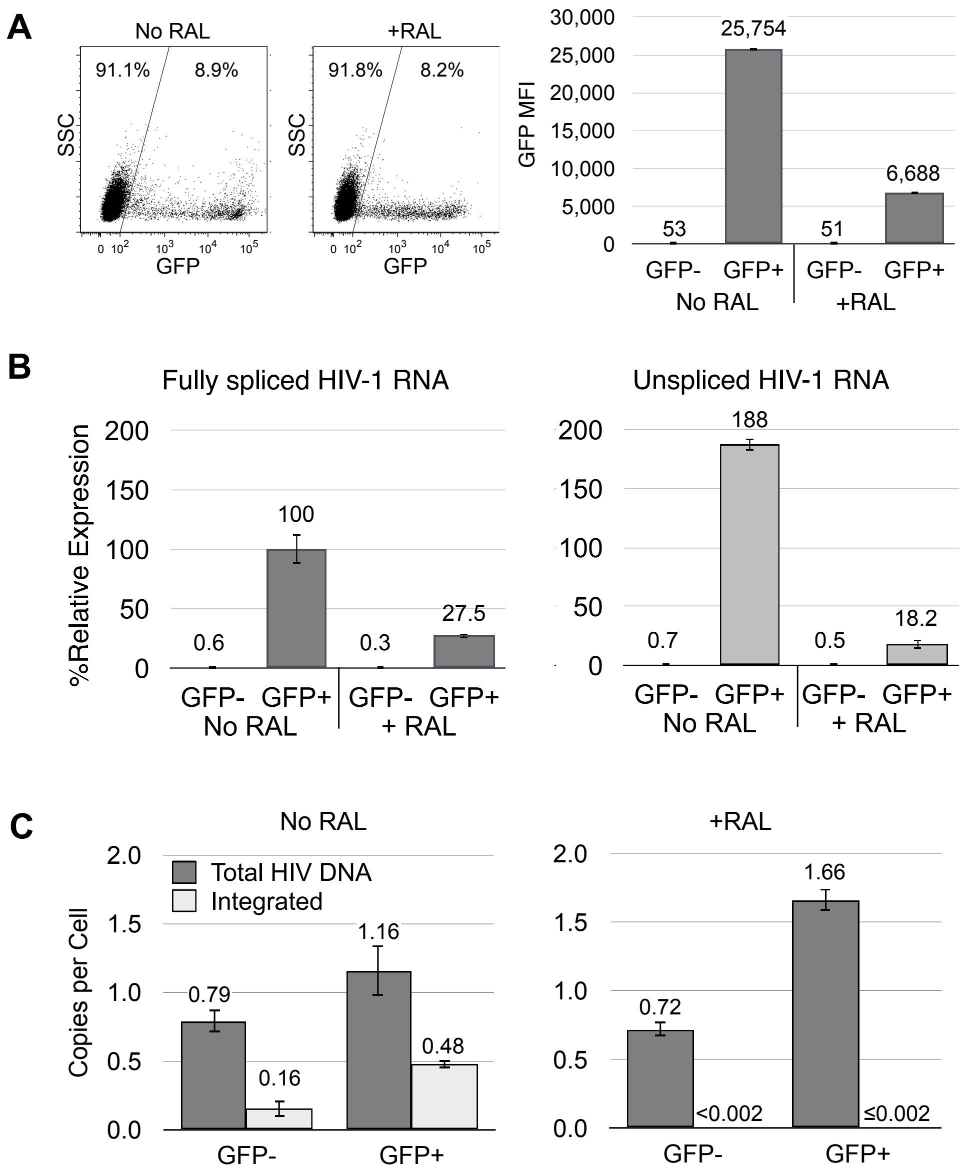

Figure S2 (supplement to Fig. 1). Protein, RNA and DNA in GFP+ and GFP- cells at day 14 post infection.

(A) HIV-GFP expression in cells at day 14 for analysis in E. Data are representative of >5 independent experiments using cells from different donors. MFI = mean fluorescence intensity of GFP.

(B) Intracellular HIV-1 RNA in sorted cells from integration permissive (No RAL) and integration inhibited (+RAL) cultures. Early Rev-independent Fully spliced RNA, and late Rev-dependent unspliced RNA were quantified by RT-qPCR (see methods). Data are normalized to Fully spliced RNA in the GFP+ No RAL cells set as 100%. Data from one of two similar experiments is shown. Standard deviations are from PCR triplicates. Non-proliferated cells were sorted for this analysis (Fig S4A-B).

(C) qPCR analysis of Total and Integrated HIV-1 DNA in cells from Fig. 1E-F. Infection in the presence of raltegravir resulted in less than 1 in 500 GFP+ cells containing an integrated provirus.

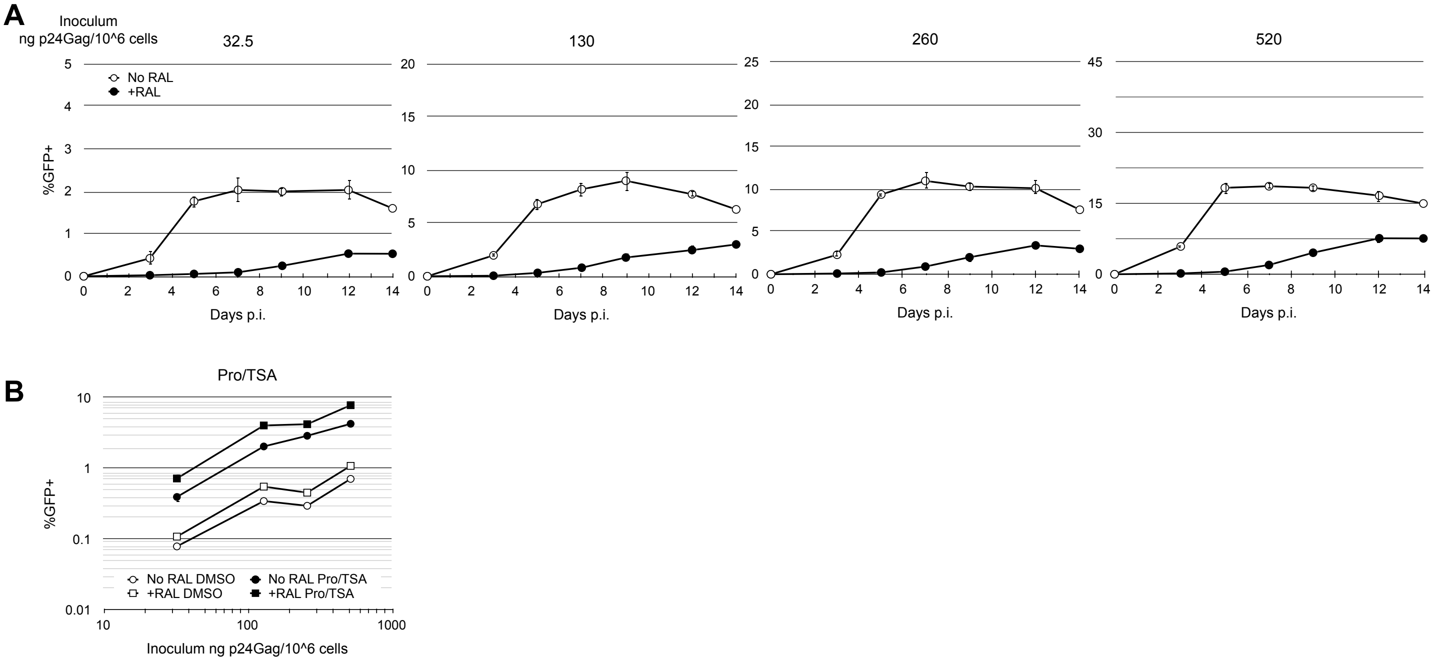

Figure S3 (supplement to Fig. 1). Lack of influence of MOI on the timing of HIV-1 gene expression and on No RAL vs. + RAL latency in resting CD4 T cells.

(A) Cells were infected as in Figure 1 except that different doses of HIV-1 were applied. Figure 1 employs 300ng p24gag per million cells. Note that the Y axis scales are adjusted to allow comparison of relative kinetics at different MOI.

(B) Relative amounts of latency for No RAL and +RAL infections remain constant with varying MOI. GFP-negative cells from each infection were sorted at Day 14 p.i. by FACS and stimulated with Pro/TSA in triplicates or DMSO. Expression of GFP one day after stimulation is shown. Data representative of 3 independent experiments with 3 different donors.

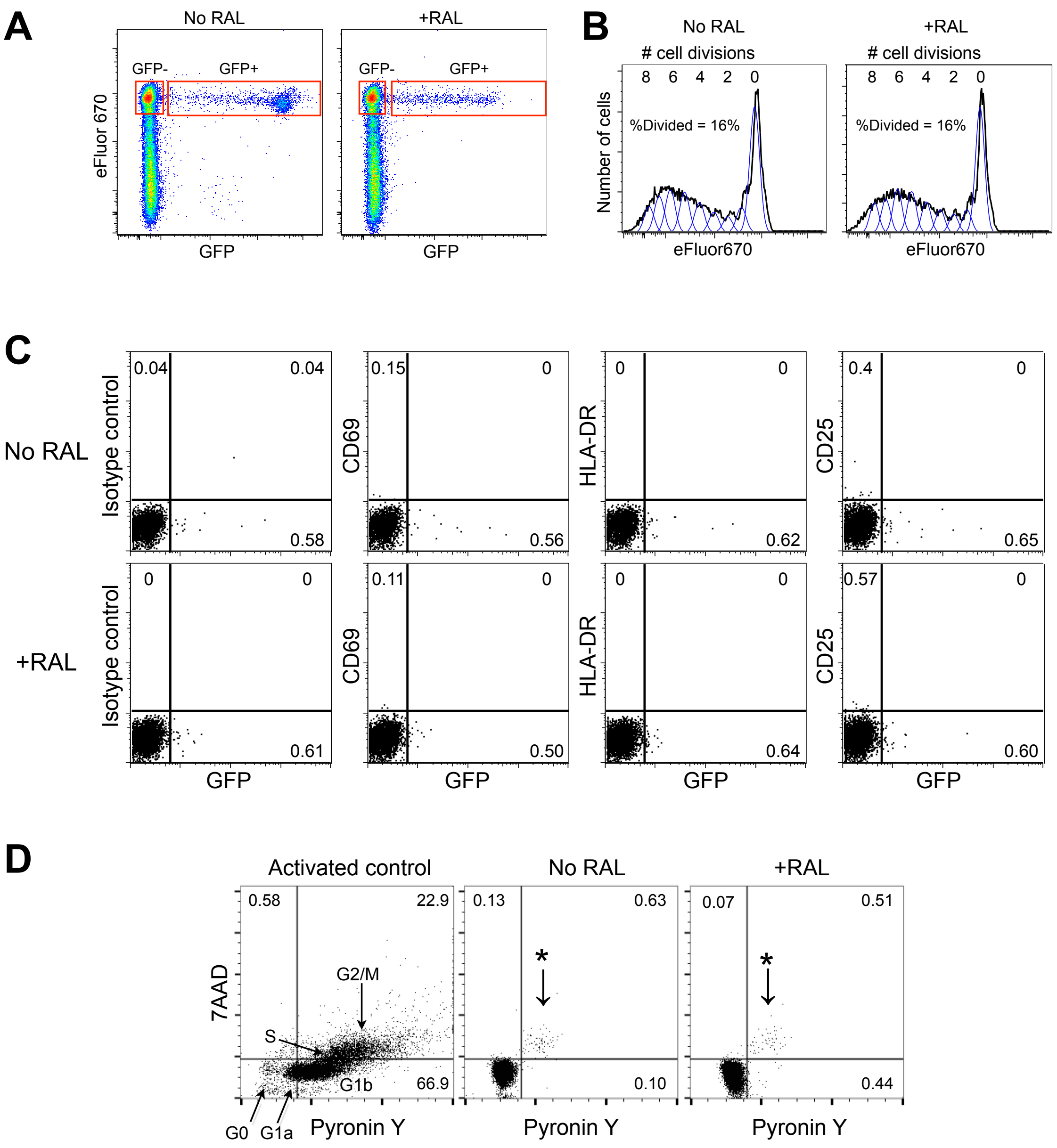

Figure S4 (supplement to Fig 1). Homeostatic proliferation of IL-4 treated cells and resting status of the non-productively infected cells.

(A) eFluor670 and GFP expression on day 14 p.i. Sorting gates are indicated by red boxes. Data are representative of >5 independent experiments using cells from different donors.

(B) Analysis of cell proliferation on the day of sorting. The FlowJo Proliferation Platform was used to calculate the percentage of cells that had divided at least once.

(C) Expression of T cell activation markers CD69, HLA-DR and CD25 on the eFluor^{hi}GFP-negative cells one day after sorting. Data are representative of three independent experiments.

(D) 7AAD-Pyronin Y staining of eFluor^{hi}GFP-negative cells one day after sort. As a positive control for cell cycle progression, uninfected CD4 T cells were activated with αCD3/CD28 beads four days prior to analysis. The arrow with * indicates cells with increased DNA content but low RNA levels that are apparently preparing for homeostatic proliferation. Data are representative of two independent experiments.

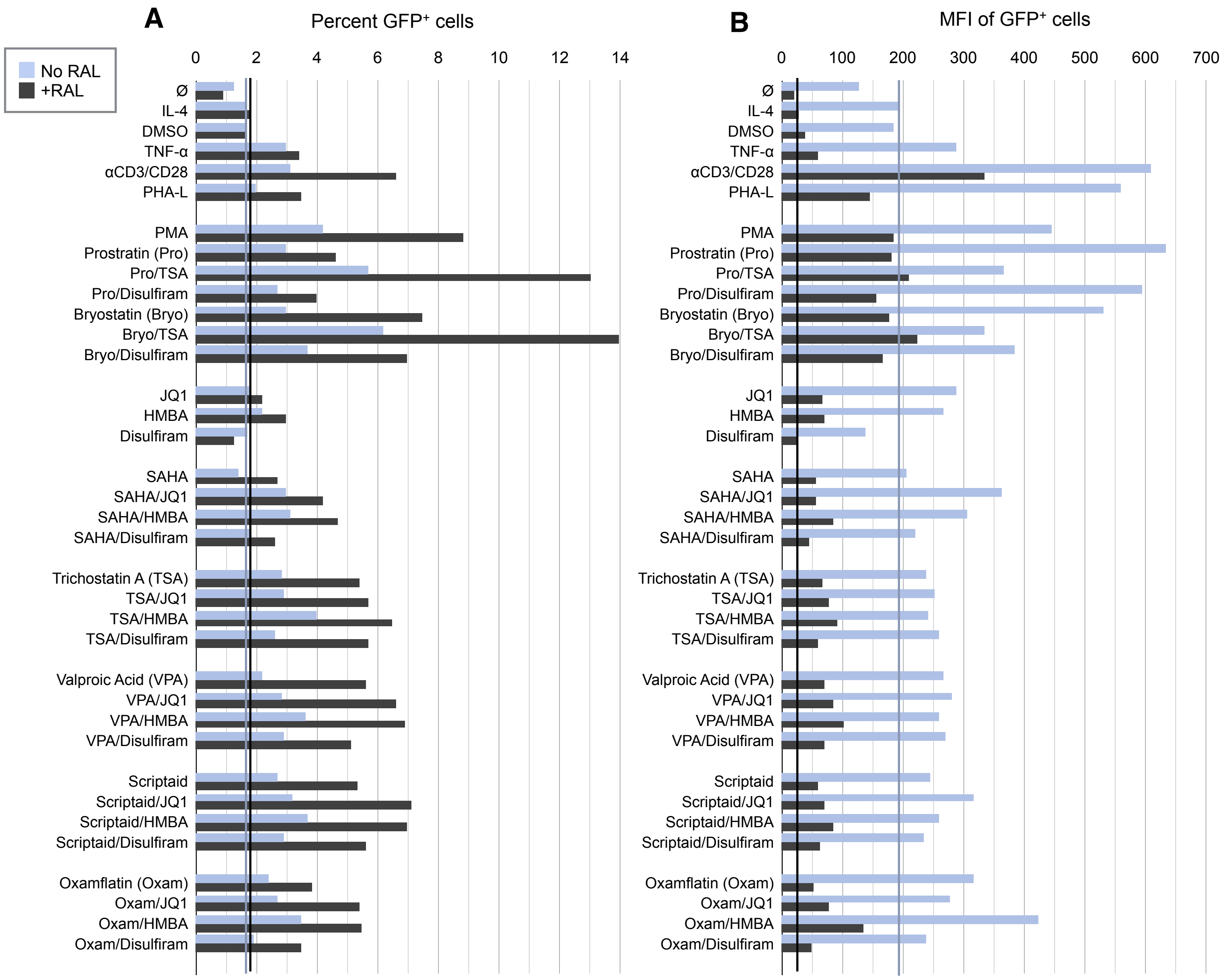

Figure S5 (supplement to Fig 2). Expanded panel of latency reversing agents in a donor different from Fig. 2. Grey and black vertical lines represent expression levels from the IL-4-only negative controls for No RAL and +RAL conditions in order to better visualize induction from latency.

(A) Percent GFP+ cells as in Fig. 2 for the expanded panel.

(B) Mean fluorescence intensity (MFI) of the GFP+ cells.

	Sorting Gate						
**Fig 4A-B: First sort**	**GFP-**	**+**	**++**	**+++**		**All Cells**	**All GFP+ cells**
% of all cells	87.6	2.8	5.4	4.2		100%	12.4%
						**Copies per cell:**	**Copies per cell:**
Total DNA copies/cell	0.8	2.7	3.7	3.1	**Total DNA:**	**1.11**	3.3
iDNA copies/cell	0.04	0.2	0.3	1.3	**Integrated DNA:**	**0.11**	0.62
Total DNA/iDNA	20.0	13.5	12.3	2.4	**Total/iDNA:**	9.9	5.3
**Fig 4C-D: Second sort**							
% of all cells	98	1.3	0.58			100%	1.88%
Total DNA copies/cell	0.7	3.6	3.4		**Total DNA:**	**0.75**	3.5
iDNA copies/cell	0.03	0.3	1.4		**Integrated DNA:**	**0.04**	0.6
Total DNA/iDNA	23.3	12.0	2.4		**Total/iDNA**	18.2	5.5

Table S1 (supplement to Fig 4). Calculations of ratios of uDNA to iDNA in sorted No RAL cells. Calculations of the ratios of integrated to total DNA in sorted cell populations for Fig. 4A and C.

Table S2 (Supplement to Fig 4). Percentage of cells with only uDNA from 4 additional experiments.

Experiment	Type	D7 p.i. %GFP+ No RAL	D7 p.i. %GFP+ +RAL	D14 p.i. %GFP+ No RAL	D14 p.i. %GFP+ +RAL	D14-15 p.i. % of GFP+ cells with only uDNA
1*	1st sort of double sort (D14 p.i.)	16	9.4	12	17	48
1*	2nd sort of double sort (D15 p.i.)	16	9.4	12	17	50
2	2nd sort of double sort (D15 p.i.)	23	9.2	20	23	55
3	Single sort (D14 p.i.)	20	2.6	13	8	10
4	Single sort (D14 p.i.)	27	3.4	10	8	9
5	1st sort of double sort (D14 p.i.)	21	5.2	17	15	17
5	2nd sort of double sort (D15 p.i.)	21	5.2	17	15	62
	Average:	20.6	6.3	14.7	14.8	35.9

*Experiment shown in Figure 4

Table S2 (Supplement to Fig 4). Percentage of cells with only uDNA from 4 additional experiments.
Results from 4 additional independent experiments utilizing different donor cells for comparison with Fig. 4.

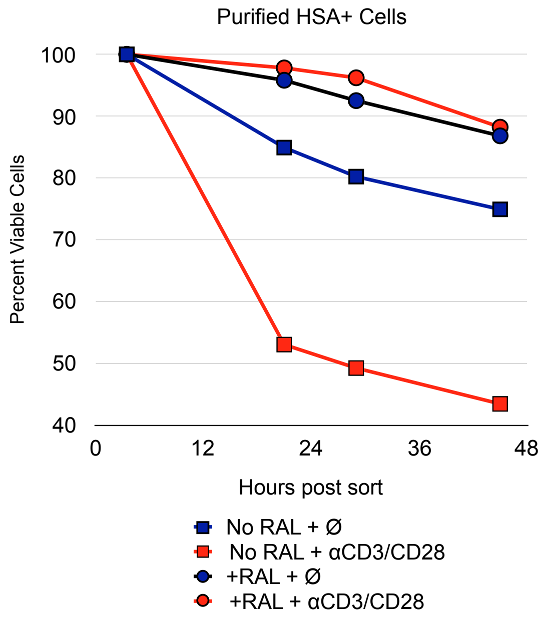

Figure S6 (supplement to Fig 5) Activation of productively infected cells accelerates death only when integration is allowed.

HSA+ cells were isolated 8 days after infection and stimulated or not with αCD3/CD28 activation beads. Viability was assessed at the indicated times by flow cytometry as in Fig. 5E. Similar results were obtained using the GFP virus and sorted GFP+ cells (not shown).

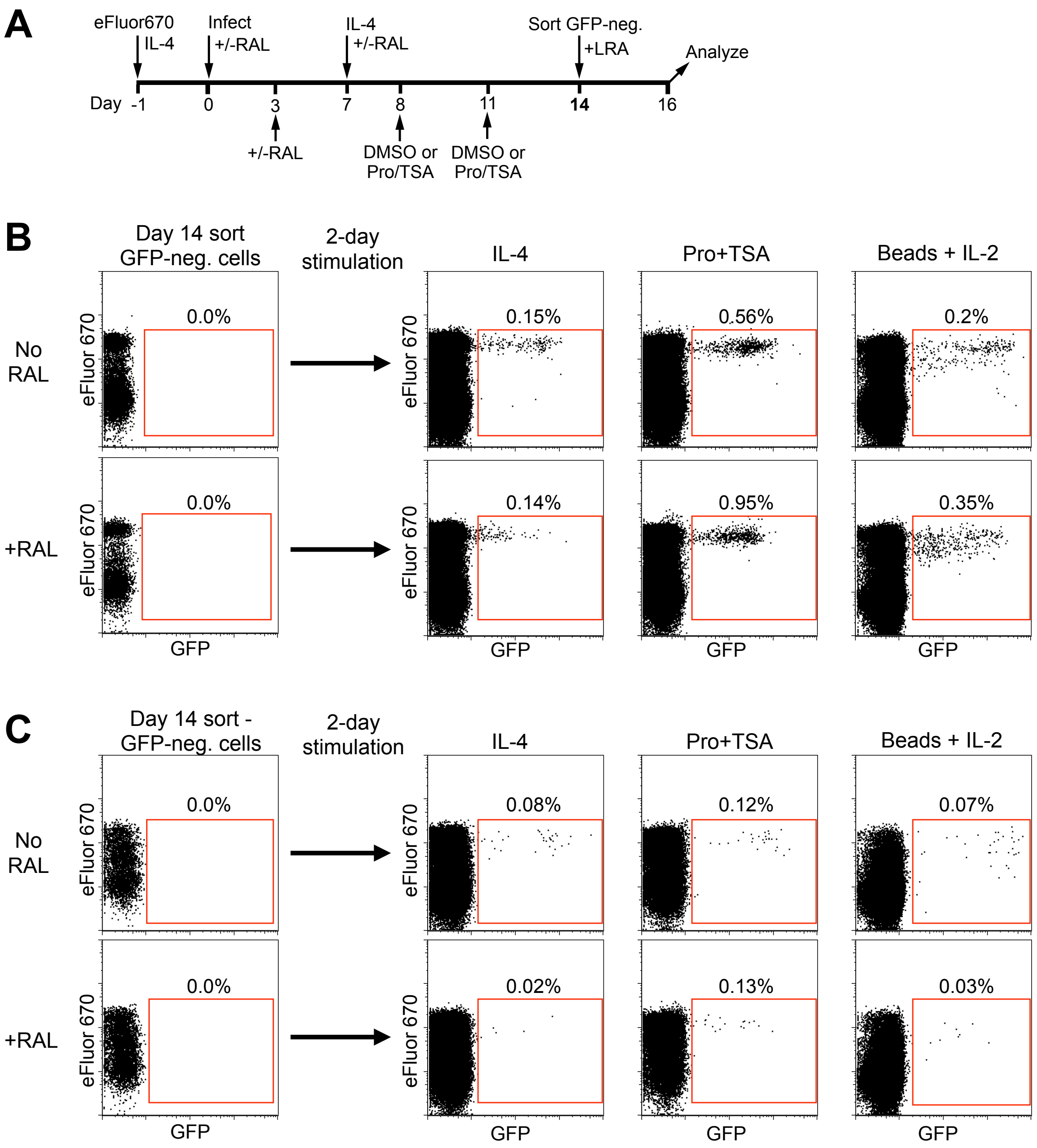

Figure S7 (supplement to Fig 6) Two rounds of stimulation by Pro/TSA did not reverse all HIV-1 latency. **(A)** Experimental design. Infected cells were treated with DMSO **(B)** or Pro/TSA **(C)** on day 8 p.i., then washed and re-treated with the same compounds on day 11 post infection. GFP-neg. cells were sorted on day 14 p.i. and treated with IL-4, Pro/TSA or αCD3/CD28 activation beads + IL-2 for 2 days before analysis. Indinavir was added at day 0 and 7 p.i. as an additional precaution against second round infection.
